# Transcriptome Analysis of Skeletal Muscle Reveals Altered Proteolytic and Neuromuscular Junction Associated Gene Expressions in a Mouse Model of Cerebral Ischemic Stroke

**DOI:** 10.3390/genes11070726

**Published:** 2020-06-30

**Authors:** Peter J. Ferrandi, Mohammad Moshahid Khan, Hector G. Paez, Christopher R. Pitzer, Stephen E. Alway, Junaith S. Mohamed

**Affiliations:** 1 Laboratory of Muscle and Nerve, Department of Diagnostic and Health Sciences, College of Health Professions, University of Tennessee Health Science Center, Memphis, TN 38163, USA; pferrand@uthsc.edu; 2 Center for Muscle, Metabolism and Neuropathology, Division of Rehabilitation Sciences, College of Health Professions, University of Tennessee Health Science Center, Memphis, TN 38163, USA; mkhan26@uthsc.edu (M.M.K.); hpaez1@uthsc.edu (H.G.P.); cpitzer1@uthsc.edu (C.R.P.); salway@uthsc.edu (S.E.A.); 3 Department of Neurology, College of Medicine, The University of Tennessee Health Science Center, Memphis, TN 38163, USA; 4 Laboratory of Muscle Biology and Sarcopenia, Department of Physical Therapy, College of Health Professions, University of Tennessee Health Science Center, Memphis, TN 38163, USA

**Keywords:** atrophy, mRNA, skeletal muscle, neuromuscular junction, stroke, transcriptomics

## Abstract

Stroke is a leading cause of mortality and long-term disability in patients worldwide. Skeletal muscle is the primary systemic target organ of stroke that induces muscle wasting and weakness, which predominantly contribute to functional disability in stroke patients. Currently, no pharmacological drug is available to treat post-stroke muscle morbidities as the mechanisms underlying post-stroke muscle wasting remain poorly understood. To understand the stroke-mediated molecular changes occurring at the transcriptional level in skeletal muscle, the gene expression profiles and enrichment pathways were explored in a mouse model of cerebral ischemic stroke via high-throughput RNA sequencing and extensive bioinformatic analyses. RNA-seq revealed that the elevated muscle atrophy observed in response to stroke was associated with the altered expression of genes involved in proteolysis, cell cycle, extracellular matrix remodeling, and the neuromuscular junction (NMJ). These data suggest that stroke primarily targets muscle protein degradation and NMJ pathway proteins to induce muscle atrophy. Collectively, we for the first time have found a novel genome-wide transcriptome signature of post-stroke skeletal muscle in mice. Our study will provide critical information to further elucidate specific gene(s) and pathway(s) that can be targeted to mitigate accountable for post-stroke muscle atrophy and related weakness.

## 1. Introduction

A cerebral vascular accident or stroke is described as a sudden interruption in the blood supply of the brain as a result of either an abrupt blockage of arteries leading to the brain (ischemic stroke) or bleeding into brain tissue when a blood vessel bursts (hemorrhagic stroke). According to the World Health Organization, stroke results in five million deaths worldwide each year, and ischemic stroke accounts for 87% of all stroke incidents [1]. Importantly, an additional five million stroke patients will experience long-term disability, reducing quality of life while contributing to the international health care burden [1]. In the United States, stroke is the fifth most common cause of death and a leading cause of long-term disability in patients [2]. In the United States, stroke affects nearly 800,000 individuals annually, and ~75% of these are first-ever strokes, whereas the remaining 25% are recurrent strokes [2]. Although stroke is often viewed as a disease of the elderly, it can occur at any age; ~10% of all strokes occur in individuals aged between 18 and 50 years [2]. 

Skeletal muscle is the main effector organ largely accounting for disability in stroke patients due to the induction of severe muscle atrophy and related weakness [3,4]. No approved pharmacological drug is available at present to treat, prevent, or minimize the loss of muscle mass and associated function in patients with stroke. This is in large part due to our poor understanding of the molecular mechanisms by which stroke initiates the muscle atrophy program. Supportive strategies such as early rehabilitative help are the only available source of treatment to improve patient recovery and functional outcomes [5,6]. However, higher muscle fatigability and lower muscle strength due to muscle atrophy, undermine the efficacy of rehabilitation [7]. Therefore, identifying the stroke-induced molecular changes at the genome level in association with skeletal muscle atrophy is indispensable for identifying therapeutic targets to reduce muscle dysfunction in stroke. The current body of literature partially attributes post-stroke muscle alterations to abnormal intraspinal processing and dysfunctional alpha motor neuron innervation [8]; however, loss of innervation is not sufficient to fully explain the loss of muscle mass and function with stroke. Additionally, stroke is often followed by reduced physical activity [3], inadequate nutrition [9], and systemic inflammation [10], which acts to exacerbate muscle dysfunction. However, there are insufficient data that describe the unique molecular changes involving transcript and/or protein expression alterations specific to skeletal muscle following stroke. Recent evidence suggests that post-stroke atrophy in skeletal muscle is largely attributed to repressed anabolism and accelerated catabolism in mice [11]. It has also been demonstrated that pharmacological inhibition of myostatin partly rescued stroke-induced skeletal muscle atrophy in a similar mouse model [12]. 

Gene expression analysis has become a central tool for studying the global mechanisms that regulate cellular homeostasis. Skeletal muscle has been widely used to study the transcriptional effects of many chronic diseases, including cancer [13] and myopathy [14]. However, the global gene expression profile of skeletal muscle following stroke has yet to be explored. The purpose of this study was to investigate gene expression changes in skeletal muscle in response to ischemic stroke to better understand the molecular origin of pathophysiological processes occurring in post-stroke skeletal muscle. It is expected that these data will provide novel insight into the mechanisms and potential targets underlying muscle wasting in stroke. The objective of this study is to determine the stroke-related gene expression profile of skeletal muscle focused on the muscle atrophy program. To achieve this, we used a transcriptome-wide RNA sequencing technique in a pre-clinical mouse model of middle cerebral artery occlusion (MCAO). The results of which demonstrated a diverse array of differentially expressed genes spanning a variety of signaling pathways involved in proteolysis and neuromuscular junction (NMJ) remodeling.

## 2. Materials and Methods

### 2.1. Animals

The C57BL/6J mice were obtained from Jackson Laboratory (Bar Harbor, ME, USA). Mice were maintained in accordance with the National Institutes of Health Guide for the Care and Use of Laboratory Animals, and the Institutional Animal Care and Use Committee of the University of Tennessee Health Science Center approved the animal protocols (ID–19-013; 2/26/2019). The mice were kept in a temperature-controlled room on a 12-h light/dark cycle, with 60% humidity, and food and water ad libitum. The mice were ~20 weeks old (~25 ± 3 g) at the time of the experiments, and 4–5 mice were used for each experiment.

### 2.2. MCAO Surgery and Tissue Collection

To induce transient cerebral ischemia, we performed MCAO in male mice as we published before [15,16]. Briefly, mice were anesthetized with 1–1.5% isoflurane (SomnoSuite, Kent Scientific Corp., Torrington, CT, USA) and maintained at 37 °C ± 1.0 body temperature using a surgical board build-in heating pad (SurgiSuite, Kent Scientific Crop.) during the entire period of the procedure. A small longitudinal incision was made along the midline of the neck. This was followed by another incision on the anterior cervical fascia being made to expose the sternocleidomastoid muscle on the right side. The anterior cervical muscle group and sternocleidomastoid muscle were separated to dissect and isolate the right common carotid artery (CCA), the external carotid artery (ECA), and the internal carotid artery (ICA). After a nick was made in the distal region of the ECA, we inserted a standardized polyamide resin glue-coated 6.0 nylon monofilament (Doccol Corp., Sharon, MA, USA) into the ECA lumen and then advanced ~9–9.5 mm in the ICA lumen to block middle cerebral artery (MCA) blood flow. After 45 min of occlusion, the suture was removed to achieve reperfusion. The ECA was cauterized after the nick to avoid any leakage of blood. After closing the incision, the mice were initially returned to the pre-warmed cage situated on a heating pad maintained at 37 °C until they recovered before they were returned to their original cages and given free access to food and water. Sham mice underwent the same surgical procedure of the MCAO mice except for the insertion of filament. The vital signs for mice that were monitored through the procedure included heart rate, body temperature, and breathing rate. Prior to sacrifice, mice were evaluated for neurological deficits by an investigator blinded to the experimental cohort. Mice were euthanized 72 h after surgery, and the brain and skeletal muscles were collected and used immediately or frozen in liquid N_2_ for future use.

### 2.3. TTC Staining and Assessment of Infarct Volume

After 72 h reperfusion, mice were anesthetized with isoflurane (4–5%) followed by euthanasia using the cervical dislocation method. The brains were isolated and placed in a brain matrice (Kent Scientific Corp.), and seven 1 mm coronal sections were made from the olfactory bulb to the cerebellum using a pre-chilled razor blade. These slices were incubated in 1.5% 2,3,5-Triphenyltetrazolium chloride (TTC) solution (Sigma, St. Louise, MO, USA) at 37 °C in an incubator for 10 min. The stained brain section images were captured with a digital scanner and transported to a computer. The infarct area of each brain slice was measured in a blinded manner using ImageJ software (National Institutes of Health). The infarct volume was calculated by Swanson’s method to correct for edema [17]. The total volumes of both contralateral and ipsilateral hemisphere, and the volumes of the striatum and cortex in both hemispheres, were measured, and the infarct percentage was calculated as % contralateral structure to avoid mismeasurement secondary to edema.

### 2.4. Assessment of Functional Outcomes

Neurologic examination and locomotor activity were measured as follows on days 1, 2, and 3. We evaluated sensorimotor performance in the mice using a neurological deficit score based on the detection of hemiparesis and abnormal posture. The pareletic hind limb of each mouse was extended gently with round-tipped forceps, and the flexor response was evaluated as 0 (normal), 1 (slight deficit), 2 (moderate deficit), or 3 (severe deficit). For assessment of posture, mice were suspended by the tail and forelimb flexion, and body twisting was evaluated as 0 (normal), 1 (slight twisting), 2 (marked twisting), or 3 (marked twisting and forelimb flexion). Spontaneous locomotor activity was measured as 0 (normal; walking straight), 1 (slight; deficit due to slightly curved walking), 2 (moderate deficit; circling), or 3 (severe deficit; not tending to walk or circling). We used the average of all the above assessment scores to generate a final neurological deficit score for each mouse.

### 2.5. RNA Sequencing and Data Analysis

Total RNA was isolated from the paralytic tibialis anterior (PTA) muscle of the stroke (ST) and corresponding TA muscle of sham (SH) mice utilizing Trizol Reagent (Thermo Fisher Scientific, Pittsburgh, PA, USA) followed by the RNeasy Plus Mini Kit (Qiagen, Germantown, MD, USA) as per manufacturer instructions. The total RNA quantity and purity were analyzed using Bioanalyzer 2100 and RNA 6000 Nano LabChip Kit (Agilent, CA, USA) and RNA integrity number (RIN) number >7.0 was used in the library preparation. Approximately 1 ug of total RNA representing a specific adipose type was used to deplete ribosomal RNA according to the Epicentre Ribo-Zero Gold Kit (Illumina, San Diego, USA). Following purification, the poly(A)- or poly(A)+ RNA fractions is fragmented into small pieces using divalent cations under elevated temperature. Then, the cleaved RNA fragments were reverse-transcribed to create the final cDNA library in accordance with the protocol for the mRNA-Seq sample preparation kit (Illumina), the average insert size for the paired-end libraries was 300 bp (±50 bp). We performed the paired-end sequencing on an Illumina Hiseq 4000 at LC Sciences (Houston, TX) following the vendor’s recommended protocol. Subsequently, Cutadapt [18] and perl scripts in house were used to remove the reads that contained adaptor contamination, low-quality bases, and undetermined bases. Then, sequence quality was verified using FastQC. We used Bowtie2 [19] and Tophat2 [20] to map reads to the mus musculus genome. The mapped reads of each sample were assembled using StringTie [21]. Then, all transcriptomes from ST and SH samples were respectively merged to reconstruct a comprehensive transcriptome using perl scripts and gffcompare [21]. After the final transcriptome was generated, StringTie [21] and Ballgown [22] was used to estimate the expression levels of all transcripts. StringTie [21] was used to perform expression level for mRNAs and lncRNAs by calculating fragments per kilobase of transcript per million mapped reads (FPKM). The differentially expressed mRNAs and lncRNAs were selected with log2 (fold change) >1 or log2 (fold change) <-1 and with statistical significance (*p*-value < 0.05) by R package Ballgown [22]. The RNA sequencing data can be found at NCBI Sequence Read Archive database under the BioProject accession number PRJNA630495 (SAMN14838464 - SAMN14838469) and SRA accession number (SRR11692190 - SRR11692195).

### 2.6. GO and KEGG Analyses

Gene ontology (GO) (http://www.geneontology.org) is a major bioinformatics initiative to unify the representation of gene and gene product attributes across all species. More specifically, the project aims to (1) maintain and develop its controlled vocabulary of gene and gene product attributes; (2) annotate genes and gene products and assimilate and disseminate annotation data; (3) provide tools for easy access to all aspects of the data provided by the project and to enable functional interpretation of experimental data using the GO, for example, via enrichment analysis. Kyoto Encyclopedia of Genes and Genomes (KEGG) (http://www.kegg.jp/) is a collection of databases dealing with genomes, biological pathways, diseases, drugs, and chemical substances. KEGG is utilized for bioinformatics research and education, including data analysis in genomics, metagenomics, metabolomics and other omics studies, modeling and simulation in systems biology, and translational research in drug development. Significant GO terms and KEGG pathway terms were both calculated by Hypergeometric equation as shown below. TB gene number = number of total genes; TS gene number = number of differentially expressed genes in total genes; B gene number = total number of gene in all GO terms; S gene number = number of differentially expressed genes in this GO term. Those GO terms with *p*-value < 0.05 were defined as significant GO terms.

$$p=1-\overset{S-1}{\underset{i=0}{\sum }}\frac{\left(\frac{B}{i}\right)\left(\frac{TB-B}{TS-i}\right)}{\left(\frac{TB}{TS}\right)}$$

### 2.7. Detailed Functional Analysis via PubMed Search Terms

An intensive search was performed on the identified differentially expressed (DE) upregulated genes via PubMed with key terms “skeletal muscle”, “atrophy”, “NMJ”, and/or “stroke”. The initial five pages of relevant search results were carefully examined, and pertinent literature reviewed to elucidate the known and potential functional gene roles related specifically to skeletal muscle homeostasis. 

### 2.8. Real-Time PCR

Real-time PCR was performed as we have described previously [23,24]. Briefly, all RNAs were treated with TURBO DNase I (ThermoFisher). One microgram of total RNA was reverse-transcribed by using SuperScript III First-Strand Synthesis Super Mix according to the manufacturer’s protocols (ThermoFisher). PCRs were performed by using SensiFAST SYBR Hi ROX Kit (Bioline, Memphis, TN) according to the manufacturer’s instructions. qPCRs were performed on an ABI 7300 Real-time PCR system (ThermoFisher Scientific). The temperature cycle profile for the qPCR reactions was 95 °C for 2 min and 40 cycles of 95 °C for 5 s and 60 °C for 15 s. Melting curve analysis was also included at one cycle of 95 °C for 1 min, 55 °C for 30 s, and 95 °C for 30 s to verify the specificity of the amplified PCR products. The relative amounts of amplified transcripts (2^−ΔCT^) were estimated by the comparative CT (ΔCT) method and normalized to an endogenous reference (GAPDH) relative to a calibrator. All PCR products were verified on agarose gel stained with ethidium bromide to discriminate between the correct amplification products and the potential primer dimers. 

## 3. Results

### 3.1. Stroke Robustly Induced Skeletal Muscle Atrophy

**Figure 1:**
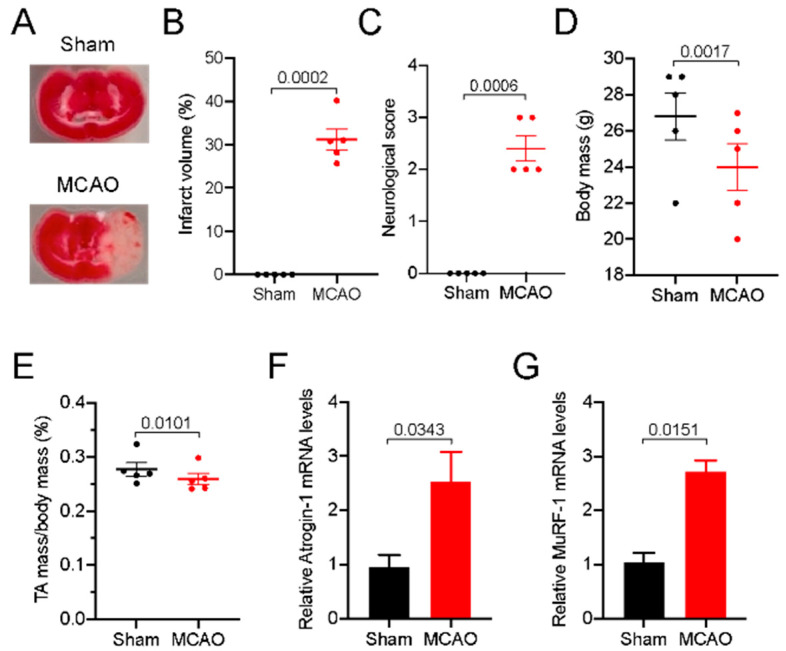
Stroke causes brain lesion, muscle wasting, and motor dysfunction. Male C57BL/6J mice were subjected to 45 min middle cerebral artery occlusion (MCAO) or sham surgery followed by 72 h reperfusion. (**A**) Representative TTC-stained 1 mm coronal brain sections of mid cortex region indicating areas of healthy (red area) and ischemic injury (white area) tissues after 72 h of reperfusion. (**B**) Size of the lesion in the ipsilateral hemisphere expressed as a % of the total contralateral hemisphere volume (*n* = 5). (**C**) Neurologic deficits were measured by the modified neurological severity score (0 is normal; *n* = 5/group). (**D**,**E**) Body mass (**D**) and PTA and CTA muscle mass normalized to body mass (**E**). (**F**,**G**) Total RNA was extracted from the PTA and CTA muscle of sham and MCAO mice (*n* = 4) and used in RT–qPCR to determine the mRNA levels of atrogin-1 (**F**) and MuRF-1 (**G**). Values in each graph indicate the mean ± SEM. PTA: Paretic tibialis anterior; CTA: the corresponding tibialis anterior.

To determine the genome-wide transcriptome profile of post-stroke skeletal muscle, we first induced cerebral ischemic stroke for 45 min using the MCAO method in mice. 45’MCAO followed by 72 h reperfusion significantly caused ~40% brain lesion in the lateral striatum and parietal cortex regions (Figure 1A,B), which severely induced motor deficit as evidenced by higher neurological score (Figure 1C). Total body weight of MCAO mice was slightly but significantly lower compared to sham mice (Figure 1D). To determine if loss of motor function could lead to muscle atrophy and weakness, we measured PTA muscle mass, which was notably lower in MCAO mice compared to the corresponding tibialis anterior (CTA) muscle of sham mice (Figure 1E). The loss of muscle mass was concomitant with the increased expressions of the well-established markers of muscle atrophy MuRF-1 (muscle RING finger 1) and MAFbx/Atrogin-1 genes in PTA muscle compared to CTA muscle (Figure 1F,G). These data indicate that 45’MCAO induced a robust ischemic brain injury that was associated with significant skeletal muscle atrophy and motor deficit and provided a strong rationale to use the muscle samples in the present study. 

### 3.2. Stroke Significantly Altered Skeletal Muscle Transcriptome Profile

**Figure 2:**
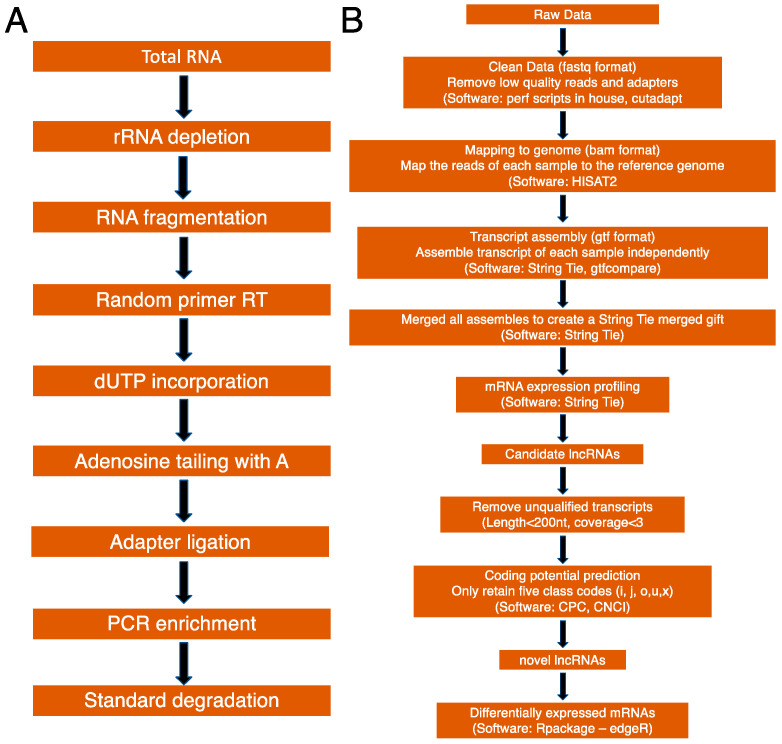
(**A**) Workflow of library preparation for sequencing and (**B**) bioinformatic analysis.

Figure 2A depicts the workflow of library preparation and RNA sequencing, while Figure 2B depicts the workflow of bioinformatic analyses. To determine if a stroke can alter the global transcriptome profile in skeletal muscle, we isolated total cellular RNAs from the PTA and CTA muscles for transcriptome sequencing (RNA-Seq). We used the RNA samples with the RIN number >7.0 for library preparation. After depletion of ribosomal RNA, followed by fragmentation with divalent cation buffers in elevated temperature, we prepared six sequencing libraries for two groups, each group containing three libraries (Group 1: sham- SH1, SH2, and SH3, and group 2: stroke- ST2, ST3, and ST4), using Illumina’s TruSeq-stranded-total-RNA-sample preparation protocol. Quality control analysis and quantification of the sequencing library were performed by Agilent Technologies 2100 Bioanalyzer High Sensitivity DNA Chip. Paired-ended sequencing was performed on Illumina’s NovaSeq 6000 sequencing system. 

Table S1 shows the database and bioinformatics software used in this study. The summary of the sequencing data is shown in Table 1. Briefly, we obtained a total of 94,947,264 (SH1), 9,3442,210 (SH2), 97,166,968 (SH3), 822,470,476 (ST2), 68,558,874 (ST3), and 740,806,190 (ST4) raw reads from the six samples. After removing adaptors, contamination, and low-quality reads, we obtained 49,031,300 (51.64%), 45,492,816 (48.69%), 43,434,064 (44.70%), 49,971,332 (60.59%), 39,457,490 (57.55%), and 44,043,614 (59.45%) clean valid reads for SH1, SH2, SH3, ST2, ST3, and ST4, respectively. We mapped these valid reads against Mus musculus genome and obtained 47,311,899 (96.49%), 43,962,039 (96.64%), 41,872,802 (96.41%), 48,017,122 (96.09%), 37,866,250 (95.97%), and 42,454,571 (96.39%) mapped reads and 40,092,540 (81.77%), 37,989,504 (83.51%), 35,870,140 (82.59%), 41,470,662 (82.99%), 32,052,577 (81.23%), and 36,442,450 (82.74%) unique reads for SH1, SH2, SH3, ST2, ST3, and ST4, respectively (Table 2).

**Figure 3:**
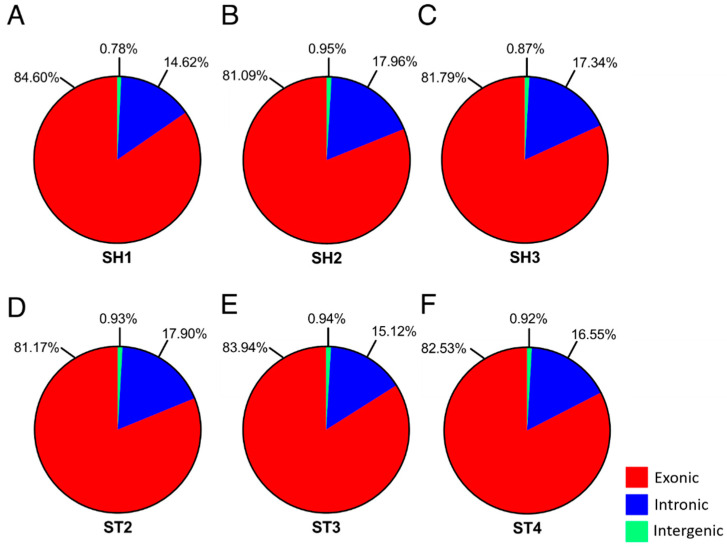
Statistics of mapped regions for SH1 (**A**), SH2 (**B**), SH3 (**C**), ST2 (**D**), ST3 (**E**), and ST4 (**F**) demonstrate that exonic regions account for the largest percentage of mapped reads in all samples, followed by intronic and intergenic regions.

**Figure 4:**
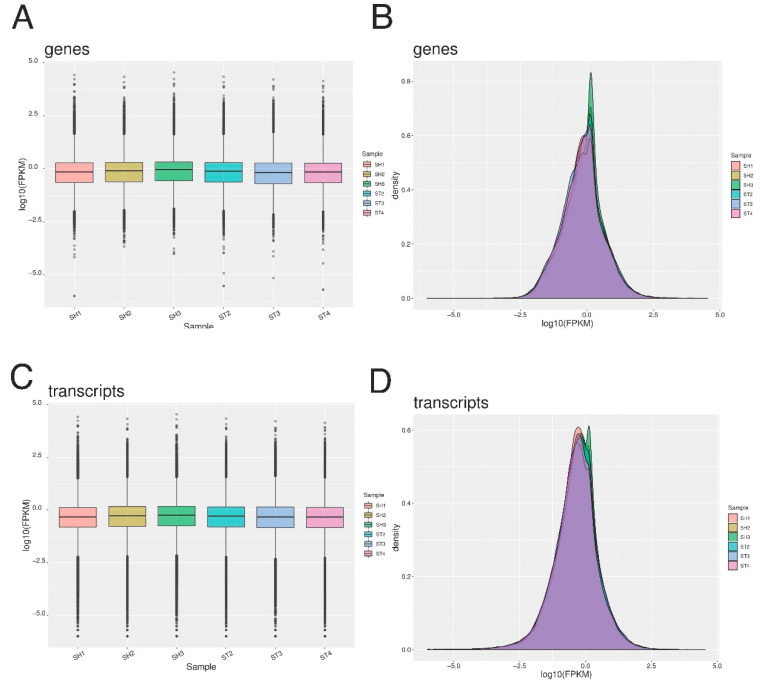
Log10 transformation of FPKM analysis for genes as box plot (**A**), and density plot (**B**), as well as for transcripts as box plot (**C**), and density plot (**D**).

As expected, most of the mapped reads are from the exon followed by intron and intergenic regions (Figure 3). Figures S1 and S2 show the localization of mRNAs on the genome as read density vs. chromosome position. Analysis by FPKM determined 86,885, 84,306, 83,971, 84,811, 77,785, and 82,780 transcripts, and 36,375, 35,766, 35,988, 36185, 33,129, and 35,113 genes for SH1, SH2, SH3, ST2, ST3, and ST4, respectively. A log10 transformation of the FPKM results for both genes and transcripts were visualized in box plot and density plot form (Figure 4). 

### 3.3. Profiling of Differentially Expressed Genes/Transcripts of Post-stroke Skeletal Muscle

**Figure 5:**
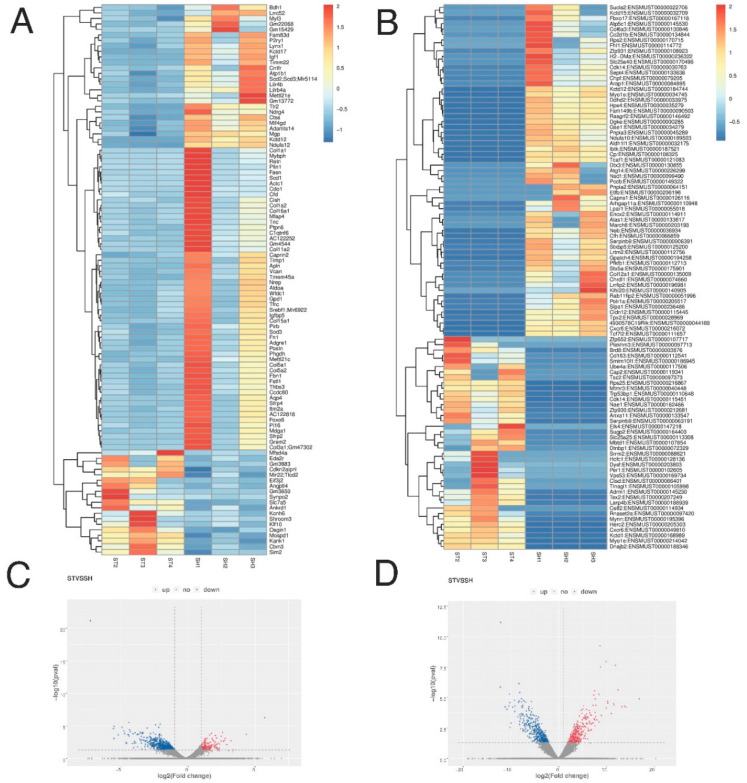
Heatmaps and volcano plots of differentially expressed genes (**A**,**C**) and transcripts (**B**,**D**).

We found 24479 differentially expressed genes with 9025 and 15,454 genes up- and downregulated, respectively in post-stroke muscle (Table S2). Among those, 1039 genes were significant at *p* ≤ 0.05, with 297 and 742 genes up- and downregulated, respectively (Table S3). Among those, 865 DE genes were significant at *p* ≤ 0.05 and exhibited a ≥ 2-fold expression change, with 173 and 692 genes up- and downregulated, respectively, in post-stroke muscle (Tables S5 and S6). Moreover, we found 78,848 unique DE transcripts, with 32,525 and 46,323 transcripts up- and downregulated, respectively (Table S4). Among those transcripts, only 1040 transcripts were significant at *p* ≤ 0.05 level, with 447 and 593 transcripts up- and downregulated, respectively (Table S4). These results are visualized via volcano plot and heat map depicting DE transcripts and genes (Figure 5).

### 3.4. GO Enrichment Analysis of Differentially Expressed Genes of Post-stroke Muscle

**Figure 6:**
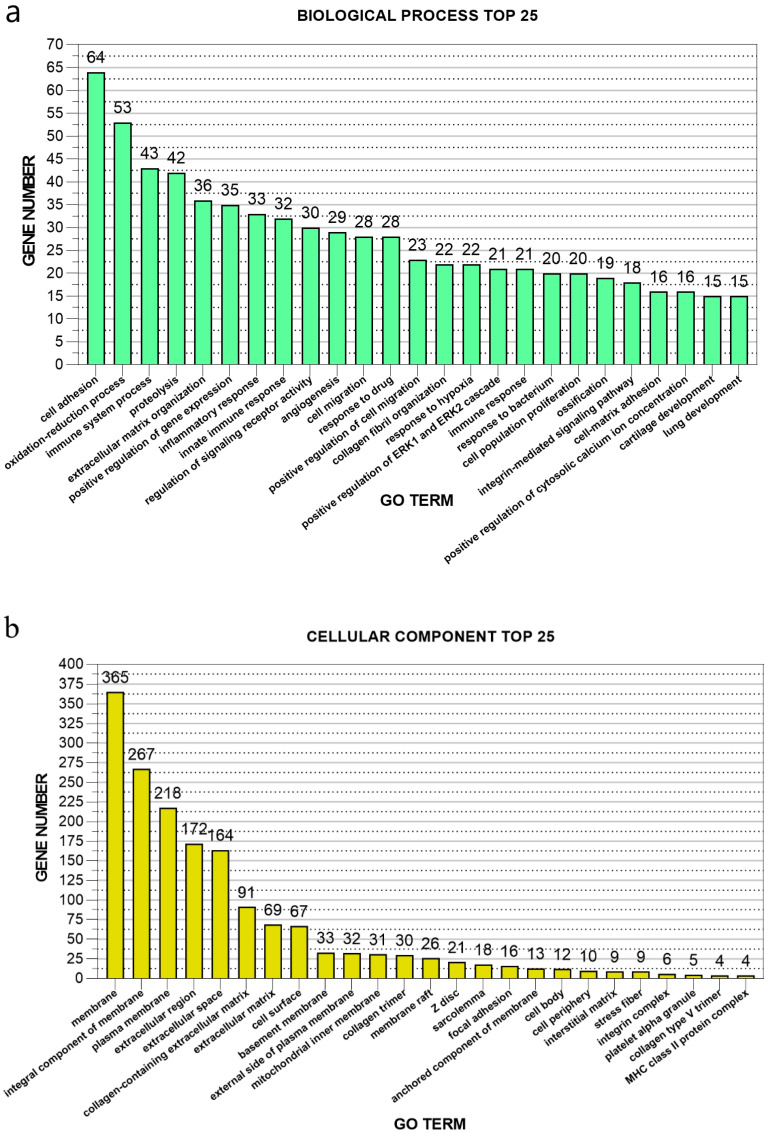

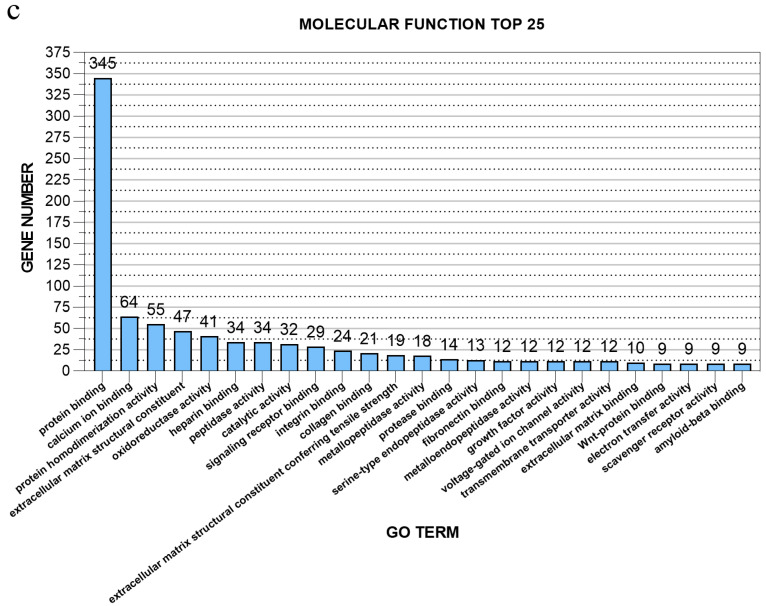
Top 25 significant gene ontology (GO) terms by gene count for Biological Process category (**a**), Cellular Component (**b**), and Molecular Function (**c**).

A total of 19,905 transcripts and 16,257 genes were annotated by GO analysis with one or more GO terms. All GO terms were divided into three subcategories: “biological process”, “cellular component,” and “molecular function”. Our GO annotation analysis resulted in 3378 (729 DE), 506 (80 DE), and 937 (215 DE) UniGenes, which were assigned terms in the biological process, cellular component, and molecular function, respectively. Most biological-process-related genes were annotated with GO terms associated with “cell adhesion”, “oxidation-reduction process”, “immune system process”, “proteolysis”, and “extracellular matrix organization” (Figure 6a). Most cellular-component-related genes were annotated with GO terms associated with “membrane” and membrane-related structure, as well as the “extracellular matrix” (Figure 6b). Most molecular-function-associated genes were annotated with GO terms associated with “protein binding”, “calcium ion binding”, “protein homodimerization activity”, “extracellular matrix structural constituents”, and “oxidoreductase activity” (Figure 6c). 

**Figure 7:**
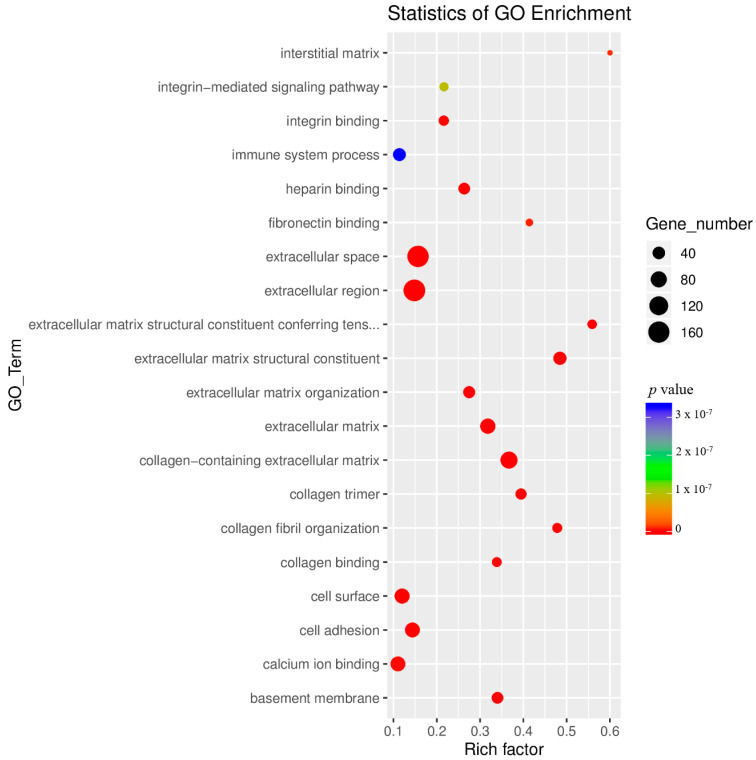
GO enrichment analysis conducted on GO terms annotated to significant differentially expressed (DE) genes across all GO categories. Rich factor is assessed by the ratio of DE genes annotated to a specific GO term to the total number of genes annotated to that GO term; a greater rich factor value indicates a greater degree of GO term enrichment.

Enrichment analysis was performed for all GO terms annotated to the significant DE genes to determine the relative degree of GO term enrichment across all categories (Figure 7). The results of our enrichment analysis demonstrated the largest degree of enrichment to GO terms associated with extracellular matrix, interstitial matrix, and collagen fibril organization (Figure 7). 

Due to the intrinsic nature of GO classification and subsequent annotation of genes/transcripts to known GO terms, a significant degree of semantic and functional overlap is frequently exhibited in GO enrichment analysis results [25,26]. Directed acyclic graphs (DAG) generated from semantic similarity analysis of the top 25 GO terms for each GO category revealed semantic similarity between annotated genes in “biological process” (Figure S3), “cellular component” (Figure S4), and “molecular function” (Figure S5). 

### 3.5. KEGG Enrichment Analysis of Differentially Expressed Genes

**Figure 8:**
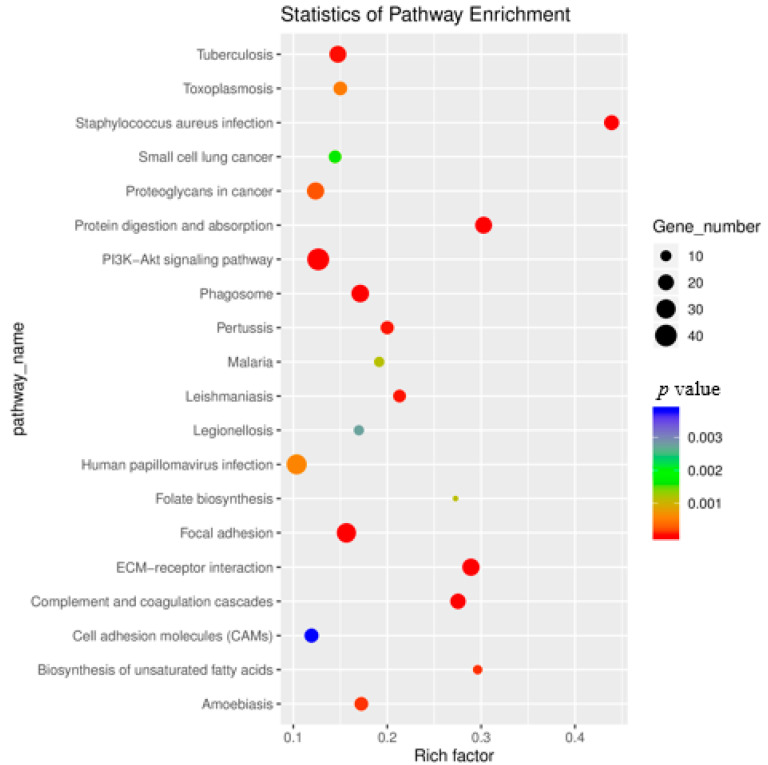
Kyoto Encyclopedia of Genes and Genomes (KEGG) enrichment analysis conducted on significant DE genes. Rich factor is assessed by the ratio of DE genes categorized in a specific KEGG pathway to the total number of genes categorized to the same KEGG pathway; a greater rich factor value indicates a greater degree of KEGG pathway enrichment.

We used KEGG enrichment analysis of DE genes to characterize their respective biological functions. A total of 6517 genes were mapped to 317 pathways. Compared to the results of our GO enrichment analysis, KEGG enrichment analysis of significant DE genes found only 52 pathways at *p* ≤ 0.05. The top 20 significantly enriched KEGG pathways, by number of annotated DE genes, *p*-value, and rich factor, were visualized as a scatterplot (Figure 8). There exist inherent differences in how KEGG annotation associates descriptive vocabulary (signaling pathways) to known genes/transcripts compared to how GO annotation associates descriptive vocabulary (ontologies) to known genes/transcripts [27]. Accordingly, we found unique associations of pathways to our DE genes via KEGG analysis. The most significantly enriched pathways by KEGG enrichment analysis were: “tuberculosis”, “toxoplasmosis”, “staphylococcus aureus infection”, “small cell lung cancer”, “proteoglycans in cancer”, “protein digestion and absorption”, and “PI3K-Akt signaling pathway”. As with the results of GO enrichment analysis, pathways associated with the extracellular matrix, compliment coagulation cascades, cell adhesion, and focal adhesion were identified as significantly enriched via KEGG enrichment analysis. Importantly, several significant DE genes were annotated by vocabulary via KEGG and GO analysis, which exhibit semantic similarity. For example, the various collagen-associated genes (e.g., Col1a1, Col1a2, Col1a3 etc.) were annotated by various pathways, including “ECM–receptor interaction”, which coincides with GO annotation conferring GO terms, such as “extracellular matrix organization”, to the same collagen-associated genes. 

### 3.6. Stroke Induced Differential Expression of Neuromuscular Junction-Associated Genes

The results of our GO and KEGG enrichment analysis indicated several annotation and pathway terms related to numerous cellular functions. Detailed analysis demonstrated stroke-induced differential expression of several NMJ- and axonal-associated genes. Sspo, Shroom3, MuSK, Chrna2, Sorbs2, and Coro6 were upregulated in response to stroke. These genes have been identified to play important roles in axonal outgrowth and/or NMJ maintenance/remodeling. Specifically, Shroom3 is involved in the Shroom3-Rho kinase signaling complex and is shown to inhibit axonal outgrowth and stroke recovery [28]; MuSK gene encodes for MuSK tyrosine kinase, which is crucial for NMJ maintenance [29,30]. Further, Cacnb1, Col4a5, P2ry1, Pmp22, and Tnc were downregulated greater than 2-fold (≤0.5 expression). These results indicate that stroke significantly influenced NMJ- and axonal-related gene expression. 

### 3.7. Differentially Expressed Ubiquitin–Proteasome Genes in Post-Stroke Muscle

The ubiquitin–proteasome pathway plays an integral role in facilitating skeletal muscle atrophy. Our initial qPCR findings indicated both Atrogin-1 (Fbxo-32) and MuRF1 (Trim63) to be significantly upregulated in response to stroke. Our sequencing analysis revealed Atrogin-1 (Fbxo-32) and MuRF1 (Trim63) to be upregulated by 1.9- and 2-fold, respectively; however *p*-values were >0.05 due to variance. Still, stroke induced differential expression of several genes related to the ubiquitin-proteasome pathway, although, Myog, Cblb, Nf32l1, Psmd8, and Trp63 were significantly upregulated ≥2-fold. Notably, Myog and Trp63 have roles in development, and both of these genes have also been shown to mediate induction of Atrogin-1 (Fbxo-32) and MuRF1 (Trim63), respectively [31,32,33]. Further, CbIb codes for a ubiquitin ligase, which targets IRS-1 for degradation, contributing to atrophy [34]. 

### 3.8. Stroke altered the Genes Associated with PI3K-Akt-mTOR Pathway

The PI3K-Akt-mTOR signaling pathway is essential for skeletal muscle protein synthesis necessary for growth and regeneration and suppression of this pathway may contribute to atrophy. The results of our sequencing analysis revealed stroke-induced differential expression of several PI3k-Akt-mTOR genes. Notably, IGF1, PIK3ap1, PIK3cg, and TLR2 were downregulated ≥2-fold (≤0.5 expression), while Mknk2 and eIF4EBP1 were upregulated ≥2-fold. eIF4EBP1 and Mknk2 expression reduce the rate of protein synthesis [35,36]. The downregulation of key PI3K-Akt activator IGF1 along with the upregulation of PI3K-Akt suppressors suggests stroke induced differential gene expression favoring reduced PI3K-Akt activation.

### 3.9. Stroke Altered p53 Pathway- and Cell-Cycle-Associated Genes

Activation of p53 occurs in response to various stress signals, such as DNA damage, oxidative stress, and activated oncogenes, regulating downstream targets related to cell cycle arrest and apoptosis. Our sequencing analysis revealed ≥2-fold upregulation of p53 associated genes Gadd45a and CdknIa. Both Gadd45a and Cdkn1a gene expression increases in response to skeletal muscle stress [37,38]. Cyclin-d genes (CCND1/2), as well as Sestrin (Sesn3) and caspase (Casp3) genes were downregulated ≥2-fold (≤0.5 expression). CCND1/2 expression contributes to myotube fate determination [39]. Sesn3 downregulation has been previously shown to correspond with disuse and aging in muscle [40]. Casp3 codes for Caspase3, a protease necessary for apoptosis; however, expression of Casp3 is also important for appropriate muscle differentiation [41,42]. Therefore, our results indicate that stroke induced differential expression of p53 associated genes in skeletal muscle, likely in response to cellular stress. 

### 3.10. Numerous Extracellular Matrix Associated Genes are Disturbed in Post-stroke Muscle

Our sequencing data demonstrate a substantial alteration to the transcriptome profile related to the ECM in muscle following stroke. There were nine collagen-specific genes shown to be significantly downregulated ≥2-fold (≤0.5 expression). Of these were Col1 and Col4, which are most important for skeletal muscle ECM formation. Additionally, ECM remodeling matrix metalloproteinases (MMP) genes such as MMP-2, as well as MMP inhibitor gene TIMP-1 were ≥2-fold (≤0.5 expression) downregulated. 

## 4. Discussion

Ischemic stroke induces widespread muscle atrophy, loss of muscle, and increased fatigue resulting in diminished quality of life and impaired recovery capability [1,43]; however, the molecular mechanisms driving these phenomena remain largely unknown. In this study, we utilized whole muscle transcriptome sequencing to identify muscle-specific transcript alterations in a pre-clinical mouse model of ischemic stroke and reperfusion. The use of an MCAO model in animals has allowed for a more robust examination of the molecular intricacies involving ischemic stroke [44]. Importantly, our RNA sequencing analyses revealed 1039 significant DE genes in mouse muscle following stroke. Of those, 173 up- and 692 downregulated genes exhibited ≥2-fold change between muscle from sham and MCAO mice. Gene ontology enrichment revealed numerous GO term annotations, with several related to cell adhesion, immune response, proteolysis, cell membrane, and extracellular matrix. The results of our KEGG enrichment revealed notable muscle-associated pathway mappings, such as “PI3K-Akt signaling pathway” and “ECM-receptor interaction”. A detailed analysis of the DE genes demonstrates that numerous ≥2-fold significant DE genes were involved in NMJ remodeling, ubiquitin–proteasome proteolysis, PI3K-Akt signaling, p53 signaling, and ECM remodeling. 

Stroke significantly affects skeletal muscle activity due to insufficient/absent impulse transmission to the NMJ that resulted in increased spasticity [8]. Importantly, models of skeletal muscle innervation dysfunction, such as sciatic denervation in mice, indicate that NMJ remodeling plays an important role in response to these innervation insults [45,46,47], and stroke could mimic many of these responses even though the structural innervation pattern has not been interrupted. Our sequencing data revealed several highly upregulated DE genes involved in NMJ maintenance and axonal outgrowth. Specifically, MuSK [29,30], Chrna2 [48], Sorbs2 [49], and Coro6 [50] were identified as encoding for proteins with known functional roles in NMJ and/or Acetylcholine receptor (AChR) maintenance and remodeling. MuSK RNA interference and gene deletion have been demonstrated to prevent appropriate adult NMJ remodeling, as well as induce the disassembly of existing NMJs [51,52]. Both Sorbs2 and Coro6 encode for proteins that play further roles, along with MuSK, in AChR clustering [49,50]. For example, Sorbs2 protein is involved in initiating the MuSK complex formation with other key proteins (e.g., MuSK/Dok-7/Crk/CrkL Complexation), thus potentiating AChR clustering at synaptic regions of the sarcolemma [49]. The encoded Coro6 protein is necessary for appropriate actin cytoskeletal anchorage and subsequent AChR clustering at the NMJ [50]. Taken together, we posit that upregulation of key NMJ-associated genes may play an integral role in response to potential loss of neural activity from the ischemic stroke. As models of ischemic stroke and denervation both share decrements in lost electric signaling through AChR, the induction of NMJ- and AChR-related genes may occur in response to reduced impulse transmission to the affected limb muscles. It is not clear to what extent the magnitude or specific gene signaling patterns that are related to NMJ dysfunction contributes to loss of muscle mass or function.

Previously, it has been shown that ischemic-stroke-induced skeletal muscle atrophy occurs in tandem with the upregulation of various genes related to the ubiquitin–ligase pathway, such as Atrogin-1 (Fbxo32) and MuRF1 (Trim63) [11,12]. The findings of our initial qPCR data further support the upregulation of these key ubiquitin–ligase genes following stroke. Although our sequencing results did not reveal Atrogin-1 (Fbxo32) or MuRF1 (Trim63) as significant DE genes due to variance between the samples, it is notable that both genes were upregulated by 1.9-fold and 2-fold, respectively, in muscles from MCAO mice as compared to sham mice. It is also interesting to note that Trp63, the transcriptional activator of the ubiquitin ligase MuRF1 (Trim63) [31], was found to be 2.01-fold upregulated in muscles from MCAO mice. Further, our sequencing analysis demonstrated significant upregulation of key ubiquitin-proteasome genes, such as Myog, Cblb, Nf32l1, and Psmd8. Although Myog is essential for muscle development [53,54], and aerobic exercise adaptations in muscle [55], it also has been shown to be elevated in the context of denervation-induced skeletal muscle atrophy, inducing various ubiquitin-ligases and contributing to muscle atrophy [32,33]. Interestingly, Myog overexpression has been shown to play a potential role in stabilization of the NMJ, helping to prevent abnormal NMJ architecture [56]. Additional work is necessary to determine the precise functions of Myog in the context of post-stroke skeletal muscle atrophy and NMJ remodeling. Nf32I1 skeletal muscle expression has been shown to increase in response to denervation contributing to atrophy [57]. As ischemic stroke and denervation both involve loss of NMJ-mediated skeletal muscle electrical activity similar to having innervation deficits, it is plausible to suggest that these genes play similar roles in stroke-induced skeletal muscle atrophy because they are both mediated by electrical activity. Cblb encodes for a ubiquitin ligase, which contributes to atrophy via degradation of IRS-1 [34]. The integral role of IRS-1 in facilitating skeletal muscle growth and regeneration is well established [58,59]; therefore, loss of IRS-1 via Cblb may contribute to stroke-induced skeletal muscle atrophy. Additionally, PSDM8 encodes for the 26S proteasome, which mediates protein degradation in skeletal muscle [60,61]. Overall, we speculate that the stroke-induced upregulation of several ubiquitin-proteasome genes in skeletal muscle suggests that the ubiquitin-proteasome system may be physiologically relevant in regulating stroke-induced muscle atrophy. 

The upregulation of proteolytic genes was accompanied by a concomitant downregulation of PI3K-Akt-mTOR associated genes. Notably, IGF1 was downregulated ≥2-fold (≤0.5 expression). IGF1 is a growth factor which robustly stimulates protein synthesis via activation of the PI3K-Akt-mTOR pathway [62]. Although the liver produces >75% of circulating IGF1, skeletal muscle also expresses IGF1 [63]. In this regard, it has been shown that overexpression of skeletal-muscle-specific IGF1 plays a protective role against skeletal muscle wasting [63]. Furthermore, stroke induced the upregulation of genes involved in PI3K-Akt suppression, such as eIF4EBP1 and Mknk2. eIF4EBP1 is a known translational inhibitor reducing protein synthesis in skeletal muscle [35,64]. Importantly, eIF4EBP1 shows a strong induction in response to skeletal muscle hindlimb unloading/disuse [64], likely contributing to disuse-associated skeletal muscle atrophy. Mknk2 encodes for mitogen-activated protein kinases-interacting kinase 2, which also reduces protein synthesis rates via inhibition of eIF4G [36]. Despite this, research on other cell-types, such as neuronal cells, suggest that inhibition of mTOR signaling may actually enhance cell survivability [65]. However, given the unique importance of mTOR signaling for skeletal muscle hypertrophy, it is plausible to suggest that stroke-induced downregulation of IGF1 and PI3K-Akt associated genes may reduce protein synthesis and contribute to skeletal muscle atrophy. 

Our analysis also demonstrated differential expression of several genes associated with the p53 pathway and cell cycle arrest. Both Gadd45a and Cdkn1a were significantly upregulated in post-stroke muscle. Interestingly, it has been previously shown that Gadd45a skeletal muscle gene expression increases during stress conditions such as illness, starvation, and disuse [66], which lead to muscle atrophy. Gadd45a encodes for the Gadd45a protein, which mediates its atrophic effects in part through interaction with mitogen-activated protein kinase kinase kinase 4 (MEKK4) [66]. It is plausible that stroke mediates stress-induced induction of Gadd45a in a similar manner to disuse, illness, and starvation. It is well-documented that denervation and DNA damage results in a gene upregulation of Cdk1a in skeletal muscle [38,67]. The encoded Cdk1a serves to play a protective role against cellular stress and DNA damage, as it arrests the cell cycle and allows for DNA repair [38]. In this regard, Cdk1a upregulation may be due to stroke-induced cellular stress, potentially to facilitate repair. Additionally, a number of p53 associated genes were downregulated in response to stroke, including CCND1/2, Sesn3, and Casp3. CCND1/2 genes encode for respective proteins that play an integral role in appropriately determining myotube fate [39]. Thus, downregulation of these genes may negatively impact skeletal muscle satellite cell differentiation because muscle stem cells can be activated presumably to increase the potential for greater anabolic transcription for regaining the lost muscle mass as an attempt to counteract muscle atrophy. Thus, loss of stem cell function in skeletal muscle after stroke could potentially contribute to poor recovery of muscle mass and associated functional improvement. Sesn3 encodes for Sestrin3, which has been shown to have a protective effect against skeletal muscle atrophy in the context of disuse and aging [40]. The significant downregulation of Sesn3 observed on our analysis suggests a potential loss of protection against muscle catabolism, possibly contributing to stroke-induced muscle atrophy. The reduced expression of Casp3 in post-stroke muscle observed in the current study may be variably interpreted. As Casp3 encodes for Caspase3, an integral apoptotic protein [41,42], downregulation may be favorable in protecting against excessive muscle atrophy. Alternatively, Caspase3 appears necessary for appropriate skeletal muscle differentiation [41,42], and thus downregulation could further impair dependent regenerative processes in post-stroke muscle. 

Lastly, our sequencing results revealed widescale transcriptome alterations related to ECM maintenance and remodeling. Several of the differentially expressed ECM genes, including Col1 and Col4, which are integral for ECM maintenance in skeletal muscle, were significantly downregulated in post-stroke muscle. We also found several MMP-related genes, such as MMP2 and TIMP1, to be significantly downregulated in post-stroke muscle. Importantly, MMP2 is needed for degradation of ECM components, which is necessary in various physiological processes [68]. In instances of skeletal muscle injury and repair, MMPs play a pivotal role in carefully degrading ECM components to allow for enhanced satellite cell migration and myonuclear function [68,69]. The ECM changes that occur in skeletal muscle following stroke are previously unknown. Here, we show a significant reduction in expression of genes which encode for ECM components, as well as genes which encode for proteins involved in ECM degradation. Although equivocal, these data demonstrate robust alterations to the ECM-associated transcriptome in skeletal muscle following stroke. This observation may also point to a disruption in post-stroke muscle satellite cell function, which has been reported to support ECM signaling [70].

The results of our transcript-wide sequencing analysis clearly demonstrate an array of differentially expressed genes in mouse skeletal muscle following transient cerebral ischemic stroke. These gene alterations occur in tandem with significant reductions in functional outcomes, such as loss of muscle mass and strength, and diminished neurological health scores. To date, no studies have performed differential transcript sequencing analysis on mouse skeletal muscle following ischemic stroke. Furthermore, limited data have been conducted to elucidate the specific molecular mechanisms underlying stroke-induced skeletal muscle atrophy. Our data demonstrate several potential molecular mechanistic targets that may be involved in the atrophic muscle response to stroke, including proteolytic pathways, NMJ maintenance/remodeling, PI3k-Akt signaling, cell cycle, and ECM maintenance/remodeling. Although these data are informative, many questions remain in regard to the effects of stroke on skeletal muscle function and health. For example, the unique ways in which stroke impacts satellite cell number and function have yet to be determined. Additional work is needed to better understand the role of stroke in satellite cell function and muscle regeneration. Moreover, future research should be conducted aiming to better understand the mechanisms underlying muscle atrophy and the detailed involvement of these pathways in post-stroke muscle. Specifically, further investigation into the role of NMJ remodeling in response to stroke-mediated innervation deficits, as well as a detailed investigation into the various proteolytic mechanisms concurrently affected, may gleam a substantially better understanding of stroke-induced skeletal muscle atrophy. 

## 5. Conclusions

The results of our RNA sequencing analysis indicate wide-scale transcriptome alterations in post-stroke muscle. Many of the identified DE genes play precise roles in muscle proteolysis, cell cycle, ECM, and NMJ remodeling. Considering the complex, multi-faceted mechanisms driving stroke-induced skeletal muscle atrophy, these results provide preliminary investigative guidance. Therefore, it may be prudent to direct future stroke research toward the pathways associated with the DE genes identified in the current study.

## Figures and Tables

**Table 1:** Summary of the transcriptome sequencing between the skeletal muscles of sham and stroke mice.

Sample	SH1	SH2	SH3	ST2	ST3	ST4
**Raw Data**						
**Read**	94,947,264	93,442,210	97,166,968	82,470,476	68,558,874	74,086,190
**Base**	14.24G	14.02G	14.58G	12.37G	10.28G	11.11G
**Valid Data**						
**Read**	49,031,300	45,492,816	43,434,064	49,971,332	39,457,490	44,043,614
**Base**	7.35G	6.82G	6.52G	7.50G	5.92G	6.61G
**Valid Ratio(reads)**	51.64	48.69	44.70	60.59	57.55	59.45
**Q20%**	99.98	99.98	99.98	99.98	99.98	99.98
**Q30%**	97.89	97.82	97.77	97.71	97.38	97.79
**GC content%**	50	49.50	49.50	50	50	49.50
**Mapped reads (%)**	47,311,899 (96.5)	43,962,039 (96.6)	41,872,802 (96.0)	48,017,122 (96.1)	37,866,250 (95.6)	42,454,571 (96.4)
**Unique Mapped reads (%)**	40,092,540 (81.8)	37,989,504 (83.5)	35,870,140 (82.6)	41,470,662 (83)	32,052,577 (81.2)	36,442,450 (82.7)
**Multi Mapped reads (%)**	7,219,359 (14.7)	5,972,535 (13.1)	6,002,662 (13.8)	6,546,460 (13.1)	5,813,673 (14.7)	6,012,121 (13.6)
**PE Mapped reads (%)**	42,315,514 (86.3)	38,786,818 (85.3)	35,419,862 (81.5)	41,386,258 (82.8)	34,974,768 (88.6)	39,438,248 (89.5)
**Reads map to sense strand (%)**	22,821,235 (46.5)	21,287,151 (46.8)	20,174,801 (46.4)	23,269,087 (46.6)	18,351,916 (46.5)	20,683,838 (47)
**Reads map to antisense strand (%)**	22,835,667 (46.6)	21,311,300 (46.8)	20,199,286 (46.5)	23,293,772 (46.6)	18,350,598 (46.5)	20,686,564 (47.0)
**Non-splice reads v**	29,191,582 (59.5)	28,846,354 (63.4)	26,973,027 (62.1)	31,573,393 (63.1)	23,944,091 (60.7)	27,383,178 (62.1)
**Splice reads (%)**	16,465,320 (33.6)	13,752,097 (30.2)	13,401,060 (30.8)	14,989,466 (30.0)	12,758,423 (32.0)	13,987,224 (31.8)
**Exon**	84.60	81.09	81.79	81.17	83.94	82.53
**Intron**	14.62	17.96	17.34	17.90	15.12	16.55
**Intergenic**	0.78	0.95	0.87	0.93	0.94	0.92

**Table 2:** Summary of statistics of gene/transcript expression between the skeletal muscles of sham and stroke mice.

Sample	SH1	SH2	SH3	ST2	ST3	ST4
**Statistics of gene expression**						
**Exp gene**	36,375	35,766	35,988	36,185	33,129	35,113
**Min.**	0.00	0.00	0.00	0.00	0.00	0.00
**1st Qu.**	0.23	0.25	0.27	0.24	0.20	0.22
**Median**	0.71	0.80	0.92	0.77	0.66	0.70
**Mean**	6.90	6.24	7.15	6.17	6.16	5.51
**3rd Qu.**	1.89	1.96	2.04	1.91	1.88	1.80
**Max.**	26,401.93	21,260.97	34,914.37	21,795.19	16,188.98	13,581.60
**Sd.**	191.68	147.86	227.22	151.37	122.48	108.64
**Sum.**	250,873.79	223,333.19	257,474.35	223,088.24	203,986.51	193,458.60
**Statistics of transcript expression**						
**Exp transcripts**	86,885	84,306	83,971	84,811	77,785	82,780
**Min.**	0.00	0.00	0.00	0.00	0.00	0.00
**1st Qu.**	0.15	0.16	0.17	0.15	0.14	0.14
**Median**	0.46	0.53	0.55	0.50	0.46	0.46
**Mean**	4.30	4.06	4.57	4.00	4.11	3.61
**3rd Qu.**	1.26	1.43	1.46	1.38	1.36	1.29
**Max.**	26,577.91	21,402.67	35,147.07	21,940.43	16,296.84	13,672.09
**Sd.**	133.78	102.12	159.33	104.79	86.48	76.47
**Sum.**	373,865.71	342,630.21	383,566.99	339,116.11	319,482.85	298,438.48
**FPKM Interval (FI) of gene expression**						
**0–0.1 FI**	4881 (13.42%)	4719 (13.19%)	4386 (12.19%)	4955 (13.69%)	4913 (14.83%)	4932 (14.05%)
**0.1–0.3 FI**	5956 (16.37%)	5445 (15.22%)	5231 (14.54%)	5601 (15.48%)	5999 (18.11%)	5877 (16.74%)
**0.3–3.57 FI**	19,698 (54.15%)	19,741 (55.19%)	20,564 (57.14%)	19,884 (54.95%)	16,796 (50.70%)	18,933 (53.92%)
**3.57–15 FI**	4306 (11.84%)	4255 (11.90%)	4303 (11.96%)	4152 (11.47%)	3811 (11.50%)	3918 (11.16%)
**15–60 FI**	1181 (3.25%)	1254 (3.51%)	1162 (3.23%)	1244 (3.44%)	1246 (3.76%)	1119 (3.19%)
**>60 FI**	353 (0.97%)	352 (0.98%)	342 (0.95%)	349 (0.96%)	364 (1.10%)	334 (0.95%)
**FPKM Interval (FI) of transcripts expression**						
**0–0.1 FI**	16,742 (19.27%)	15,316 (18.17%)	14,704 (17.51%)	15,921 (18.77%)	15,449 (19.86%)	16,322 (19.72%)
**0.1–0.3 FI**	17,136 (19.72%)	15,413 (18.28%)	15,320 (18.24%)	16,014 (18.88%)	15,489 (19.91%)	16,644 (20.11%)
**0.3–3.57 FI**	44,076 (50.73%)	44,123 (52.34%)	44,891 (53.46%)	43,671 (51.49%)	37,956 (48.80%)	41,312 (49.91%)
**3.57–15 FI**	6552 (7.54%)	6986 (8.29%)	6703 (7.98%)	6783 (8.00%)	6415 (8.25%)	6225 (7.52%)
**15–60 FI**	1814 (2.09%)	1888 (2.24%)	1784 (2.12%)	1866 (2.20%)	1894 (2.43%)	1740 (2.10%)
**>60 FI**	565 (0.65%)	580 (0.69%)	569 (0.68%)	556 (0.66%)	582 (0.75%)	537 (0.65%)
**Transcript expression coverage**						
**0–1**	29.61%	29.87%	30.08%	29.25%	34.54%	32.32%
**2–5**	39.13%	38.06%	37.86%	37.82%	36.91%	38.56%
**6–10**	14.12%	14.99%	15.89%	15.08%	12.58%	13.45%
**11–15**	4.80%	4.83%	4.93%	5.16%	4.43%	4.44%
**16–20**	2.66%	2.73%	2.58%	2.75%	2.36%	2.40%
**21–25**	1.71%	1.74%	1.64%	1.75%	1.62%	1.54%
**26–30**	1.18%	1.16%	1.08%	1.27%	1.16%	1.04%
**>30**	6.79%	6.62%	5.94%	6.92%	6.40%	6.25%

## Supplementary Materials

The following are available online at https://www.mdpi.com/2073-4425/11/7/726/s1, Figure S1: Mapped Read Density vs. Chromosome Position of Sham Muscles; Figure S2: Mapped Read Density vs. Chromosome Position of Post-Stroke Muscles; Figure S3: DAG Analysis of GO Terms for “biological process”; Figure S4: DAG Analysis of GO Terms for “cellular component”; Figure S5: DAG Analysis of GO Terms for “molecular function”; Table S1: Bioinformatic software details; Table S2: Full list of differentially expressed genes; Table S3: Differentially expressed genes with *p* < 0.05; Table S4: Full list of differentially expressed transcripts; Table S5: List of up-regulated genes that were significant at *p* ≤ 0.05 and exhibited a ≥ 2-fold expression change; Table S6: List of down-regulated genes that were significant at *p* ≤ 0.05 and exhibited a ≥ 2-fold expression change.

## References

[1] Feigin V.L., Roth G.A., Naghavi M., Parmar P., Krishnamurthi R., Chugh S., Mensah G.A., Norrving B., Shiue I., Ng M. (2016). Global burden of stroke and risk factors in 188 countries, during 1990–2013: A systematic analysis for the Global Burden of Disease Study 2013. Lancet Neurol..

[2] Benjamin E.J., Virani S.S., Callaway C.W., Chamberlain A.M., Chang A.R., Cheng S., Chiuve S.E., Cushman M., Delling F.N., Deo R. (2018). Heart Disease and Stroke Statistics-2018 Update: A Report From the American Heart Association. Circulation.

[3] Scherbakov N., Von Haehling S., Anker S.D., Dirnagl U., Doehner W. (2013). Stroke induced Sarcopenia: Muscle wasting and disability after stroke. Int. J. Cardiol..

[4] English C., McLennan H., Thoirs K., Coates A., Bernhardt J. (2010). Loss of skeletal muscle mass after stroke: A systematic review. Int. J. Stroke.

[5] Dobkin B.H. (2008). Training and exercise to drive poststroke recovery. Nat. Clin. Pract. Neurol..

[6] Saunders D.H., Greig C.A., Young A., Mead G.E. (2004). Physical fitness training for stroke patients. Stroke.

[7] Dobkin B.H. (2005). Clinical practice. Rehabilitation after stroke. N. Engl. J. Med..

[8] Li S., Francisco G.E. (2015). New insights into the pathophysiology of post-stroke spasticity. Front. Hum. Neurosci..

[9] Bouziana S.D., Tziomalos K. (2011). Malnutrition in patients with acute stroke. J. Nutr. Metab..

[10] Anrather J., Iadecola C. (2016). Inflammation and stroke: An overview. Neurotherapeutics.

[11] Desgeorges M.M., Devillard X., Toutain J., Divoux D., Castells J., Bernaudin M., Touzani O., Freyssenet D.G. (2015). Molecular mechanisms of skeletal muscle atrophy in a mouse model of cerebral ischemia. Stroke.

[12] Desgeorges M.M., Devillard X., Toutain J., Castells J., Divoux D., Arnould D.F., Haqq C., Bernaudin M., Durieux A.-C., Touzani O. (2017). Pharmacological inhibition of myostatin improves skeletal muscle mass and function in a mouse model of stroke. Sci. Rep. UK.

[13] Freire P.P., Fernandez G.J., Cury S.S., de Moraes D., Oliveira J.S., de Oliveira G., Dal-Pai-Silva M., dos Reis P.P., Carvalho R.F. (2019). The Pathway to Cancer Cachexia: MicroRNA-Regulated Networks in Muscle Wasting Based on Integrative Meta-Analysis. Int. J. Mol. Sci..

[14] Llano-Diez M., Fury W., Okamoto H., Bai Y., Gromada J., Larsson L. (2019). RNA-sequencing reveals altered skeletal muscle contraction, E3 ligases, autophagy, apoptosis, and chaperone expression in patients with critical illness myopathy. Skelet. Muscle.

[15] Khan M.M., Gandhi C., Chauhan N., Stevens J.W., Motto D.G., Lentz S.R., Chauhan A.K. (2012). Alternatively-Spliced Extra Domain a of Fibronectin Promotes Acute Inflammation and Brain Injury After Cerebral Ischemia in Mice. Stroke.

[16] Khan M.M., Motto D.G., Lentz S.R., Chauhan A.K. (2012). ADAMTS13 reduces VWF-mediated acute inflammation following focal cerebral ischemia in mice. J. Thromb. Haemost.

[17] Swanson R.A., Morton M.T., Tsao-Wu G., Savalos R.A., Davidson C., Sharp F.R. (1990). A semiautomated method for measuring brain infarct volume. J. Cereb. Blood Flow Metab..

[18] Martin M. (2011). Cutadapt removes adapter sequences from high-throughput sequencing reads. EMBnet. J..

[19] Langmead B., Salzberg S.L. (2012). Fast gapped-read alignment with Bowtie 2. Nat. Methods.

[20] Kim D., Pertea G., Trapnell C., Pimentel H., Kelley R., Salzberg S.L. (2013). TopHat2: Accurate alignment of transcriptomes in the presence of insertions, deletions and gene fusions. Genome Biol..

[21] Pertea M., Pertea G.M., Antonescu C.M., Chang T.-C., Mendell J.T., Salzberg S.L. (2015). StringTie enables improved reconstruction of a transcriptome from RNA-seq reads. Nat. Biotechnol..

[22] Frazee A.C., Pertea G., Jaffe A.E., Langmead B., Salzberg S.L., Leek J.T. (2015). Ballgown bridges the gap between transcriptome assembly and expression analysis. Nat. Biotechnol..

[23] Mohamed J.S., Lopez M.A., Boriek A.M. (2010). Mechanical stretch up-regulates microRNA-26a and induces human airway smooth muscle hypertrophy by suppressing glycogen synthase kinase-3beta. J. Biol. Chem..

[24] Mohamed J.S., Lopez M.A., Cox G.A., Boriek A.M. (2010). Anisotropic regulation of Ankrd2 gene expression in skeletal muscle by mechanical stretch. FASEB J. Off. Publ. Fed. Am. Soc. Exp. Biol..

[25] Pesquita C., Dessimoz C., Škunca N. (2017). Semantic Similarity in the Gene Ontology. The Gene Ontology Handbook.

[26] Liu W., Liu J., Rajapakse J.C. (2018). Gene Ontology Enrichment Improves Performances of Functional Similarity of Genes. Sci. Rep..

[27] Chowdhury S., Sarkar R.R. (2015). Comparison of human cell signaling pathway databases—Evolution, drawbacks and challenges. Database.

[28] Dickson H.M., Wilbur A., Reinke A.A., Young M.A., Vojtek A.B. (2015). Targeted inhibition of the Shroom3-Rho kinase protein-protein interaction circumvents Nogo66 to promote axon outgrowth. BMC Neurosci..

[29] Herbst R. (2020). MuSK function during health and disease. Neurosci. Lett..

[30] Burden S.J., Yumoto N., Zhang W. (2013). The role of MuSK in synapse formation and neuromuscular disease. Cold Spring Harb. Perspect. Biol..

[31] von Grabowiecki Y., Abreu P., Blanchard O., Palamiuc L., Benosman S., Meriaux S., Devignot V., Gross I., Mellitzer G., de Aguilar J.L.G. (2016). Transcriptional activator TAp63 is upregulated in muscular atrophy during ALS and induces the pro-atrophic ubiquitin ligase Trim63. Elife.

[32] Macpherson P.C.D., Wang X., Goldman D. (2011). Myogenin regulates denervation-dependent muscle atrophy in mouse soleus muscle. J. Cell Biochem..

[33] Moresi V., Williams A.H., Meadows E., Flynn J.M., Potthoff M.J., McAnally J., Shelton J.M., Backs J., Klein W.H., Richardson J.A. (2010). Myogenin and class II HDACs control neurogenic muscle atrophy by inducing E3 ubiquitin ligases. Cell.

[34] Kawai N., Hirasaka K., Maeda T., Haruna M., Shiota C., Ochi A., Abe T., Kohno S., Ohno A., Teshima-Kondo S. (2015). Prevention of skeletal muscle atrophy in vitro using anti-ubiquitination oligopeptide carried by atelocollagen. Biochim. Biophys. Acta BBA Mol. Cell Res..

[35] Yanagiya A., Suyama E., Adachi H., Svitkin Y.V., Aza-Blanc P., Imataka H., Mikami S., Martineau Y., Ronai Z.e.A., Sonenberg N. (2012). Translational homeostasis via the mRNA cap-binding protein, eIF4E. Mol. Cell.

[36] Hu S.-I., Katz M., Chin S., Qi X., Cruz J., Ibebunjo C., Zhao S., Chen A., Glass D.J. (2012). MNK2 Inhibits eIF4G Activation Through a Pathway Involving Serine-Arginine–Rich Protein Kinase in Skeletal Muscle. Sci. Signal..

[37] Ebert S.M., Dyle M.C., Kunkel S.D., Bullard S.A., Bongers K.S., Fox D.K., Dierdorff J.M., Foster E.D., Adams C.M. (2012). Stress-induced skeletal muscle Gadd45a expression reprograms myonuclei and causes muscle atrophy. J. Biol. Chem..

[38] Cazzalini O., Scovassi A.I., Savio M., Stivala L.A., Prosperi E. (2010). Multiple roles of the cell cycle inhibitor p21CDKN1A in the DNA damage response. Mutat. Res. Rev. Mutat. Res..

[39] Kong D., He M., Yang L., Zhou R., Yan Y.-Q., Liang Y., Teng C.-B. (2019). MiR-17 and miR-19 cooperatively promote skeletal muscle cell differentiation. Cell. Mol. Life Sci..

[40] Segalés J., Perdiguero E., Serrano A.L., Sousa-Victor P., Ortet L., Jardí M., Budanov A.V., Garcia-Prat L., Sandri M., Thomson D.M. (2020). Sestrin prevents atrophy of disused and aging muscles by integrating anabolic and catabolic signals. Nat. Commun..

[41] Fernando P., Kelly J.F., Balazsi K., Slack R.S., Megeney L.A. (2002). Caspase 3 activity is required for skeletal muscle differentiation. Proc. Natl. Acad. Sci. USA.

[42] Larsen B.D., Rampalli S., Burns L.E., Brunette S., Dilworth F.J., Megeney L.A. (2010). Caspase 3/caspase-activated DNase promote cell differentiation by inducing DNA strand breaks. Proc. Natl. Acad. Sci. USA.

[43] Hunnicutt J.L., Gregory C.M. (2017). Skeletal muscle changes following stroke: A systematic review and comparison to healthy individuals. Top. Stroke Rehabil..

[44] Chiang T., Messing R.O., Chou W.-H. (2011). Mouse model of middle cerebral artery occlusion. J. Vis. Exp. JoVE.

[45] Yampolsky P., Pacifici P.G., Witzemann V. (2010). Differential muscle-driven synaptic remodeling in the neuromuscular junction after denervation. Eur. J. Neurosci..

[46] Yee W.C., Pestronk A. (1987). Mechanisms of postsynaptic plasticity: Remodeling of the junctional acetylcholine receptor cluster induced by motor nerve terminal outgrowth. J. Neurosci..

[47] Castets P., Rion N., Théodore M., Falcetta D., Lin S., Reischl M., Wild F., Guérard L., Eickhorst C., Brockhoff M. (2019). mTORC1 and PKB/Akt control the muscle response to denervation by regulating autophagy and HDAC4. Nat. Commun..

[48] Pedersen J.E., Bergqvist C.A., Larhammar D. (2019). Evolution of vertebrate nicotinic acetylcholine receptors. BMC Evol. Biol..

[49] Hallock P.T., Chin S., Blais S., Neubert T.A., Glass D.J. (2015). Sorbs1 and -2 Interact with CrkL and Are Required for Acetylcholine Receptor Cluster Formation. Mol. Cell. Biol..

[50] Chen Y., Ip F.C., Shi L., Zhang Z., Tang H., Ng Y.P., Ye W.-C., Fu A.K., Ip N.Y. (2014). Coronin 6 regulates acetylcholine receptor clustering through modulating receptor anchorage to actin cytoskeleton. J. Neurosci..

[51] Kong X.C., Barzaghi P., Ruegg M.A. (2004). Inhibition of synapse assembly in mammalian muscle in vivo by RNA interference. EMBO Rep..

[52] Hesser B.A., Henschel O., Witzemann V. (2006). Synapse disassembly and formation of new synapses in postnatal muscle upon conditional inactivation of MuSK. Mol. Cell. Neurosci..

[53] Nabeshima Y., Hanaoka K., Hayasaka M., Esumi E., Li S., Nonaka I., Nabeshima Y. (1993). Myogenin gene disruption results in perinatal lethality because of severe muscle defect. Nature.

[54] Hasty P., Bradley A., Morris J.H., Edmondson D.G., Venuti J.M., Olson E.N., Klein W.H. (1993). Muscle deficiency and neonatal death in mice with a targeted mutation in the myogenin gene. Nature.

[55] Siu P.M., Donley D.A., Bryner R.W., Alway S.E. (2004). Myogenin and oxidative enzyme gene expression levels are elevated in rat soleus muscles after endurance training. J. Appl. Physiol..

[56] Chen H.H., Tsai L.K., Liao K.Y., Wu T.C., Huang Y.H., Huang Y.C., Chang S.W., Wang P.Y., Tsao Y.P., Chen S.L. (2018). Muscle-restricted nuclear receptor interaction protein knockout causes motor neuron degeneration through down-regulation of myogenin at the neuromuscular junction. J. Cachexia Sarcopenia Muscle.

[57] Furuya N., Ikeda S.-I., Sato S., Soma S., Ezaki J., Trejo J.A.O., Takeda-Ezaki M., Fujimura T., Arikawa-Hirasawa E., Tada N. (2014). PARK2/Parkin-mediated mitochondrial clearance contributes to proteasome activation during slow-twitch muscle atrophy via NFE2L1 nuclear translocation. Autophagy.

[58] Long Y.C., Cheng Z., Copps K.D., White M.F. (2011). Insulin receptor substrates Irs1 and Irs2 coordinate skeletal muscle growth and metabolism via the Akt and AMPK pathways. Mol. Cell. Biol..

[59] Sarah Eckstein S., Weigert C., Lehmann R. (2017). Divergent roles of IRS (insulin receptor substrate) 1 and 2 in liver and skeletal muscle. Curr. Med. Chem..

[60] Lee D., Takayama S., Goldberg A.L. (2018). ZFAND5/ZNF216 is an activator of the 26S proteasome that stimulates overall protein degradation. Proc. Natl. Acad. Sci. USA.

[61] Heng A., Ventadour S., Jarzaguet M., Pouch-Pelissier M., Guezennec C., Bigard X., Attaix D., Taillandier D. (2008). Coordinate expression of the 19S regulatory complex and evidence for ubiquitin-dependent telethonin degradation in the unloaded soleus muscle. Int. J. Biochem. Cell Biol..

[62] Schiaffino S., Mammucari C. (2011). Regulation of skeletal muscle growth by the IGF1-Akt/PKB pathway: Insights from genetic models. Skelet. Muscle.

[63] Song Y.-H., Li Y., Du J., Mitch W.E., Rosenthal N., Delafontaine P. (2005). Muscle-specific expression of IGF-1 blocks angiotensin II–induced skeletal muscle wasting. J. Clin. Investig..

[64] Llano-Diez M., Gustafson A.-M., Olsson C., Goransson H., Larsson L. (2011). Muscle wasting and the temporal gene expression pattern in a novel rat intensive care unit model. BMC Genom..

[65] Guo W., Qian L., Zhang J., Zhang W., Morrison A., Hayes P., Wilson S., Chen T., Zhao J. (2011). Sirt1 overexpression in neurons promotes neurite outgrowth and cell survival through inhibition of the mTOR signaling. J. Neurosci. Res..

[66] Bullard S.A., Seo S., Schilling B., Dyle M.C., Dierdorff J.M., Ebert S.M., DeLau A.D., Gibson B.W., Adams C.M. (2016). Gadd45a Protein Promotes Skeletal Muscle Atrophy by Forming a Complex with the Protein Kinase MEKK4. J. Biol. Chem..

[67] Magnusson C., Svensson A., Christerson U., Tågerud S. (2005). Denervation-induced alterations in gene expression in mouse skeletal muscle. Eur. J. Neurosci..

[68] Chen X., Li Y. (2009). Role of matrix metalloproteinases in skeletal muscle: Migration, differentiation, regeneration and fibrosis. Cell Adh. Migr..

[69] Yeghiazaryan M., Żybura-Broda K., Cabaj A., Włodarczyk J., Sławińska U., Rylski M., Wilczyński G.M. (2012). Fine-structural distribution of MMP-2 and MMP-9 activities in the rat skeletal muscle upon training: A study by high-resolution in situ zymography. Histochem. Cell Biol..

[70] Fry C.S., Kirby T.J., Kosmac K., McCarthy J.J., Peterson C.A. (2017). Myogenic Progenitor Cells Control Extracellular Matrix Production by Fibroblasts during Skeletal Muscle Hypertrophy. Cell Stem Cell.

