# Supplementary material for: Transcriptome Analysis of Skeletal Muscle Reveals Altered Proteolytic and Neuromuscular Junction Associated Gene Expressions in a Mouse Model of Cerebral Ischemic Stroke
Source: Genes (Basel). 2020 Jun 30;11(7):726. doi: 10.3390/genes11070726 (PMC7397267; doi:10.3390/genes11070726)
Supplement: Supplementary file 1 [file genes-11-00726-s001.zip › Supplemental Figures.pdf]

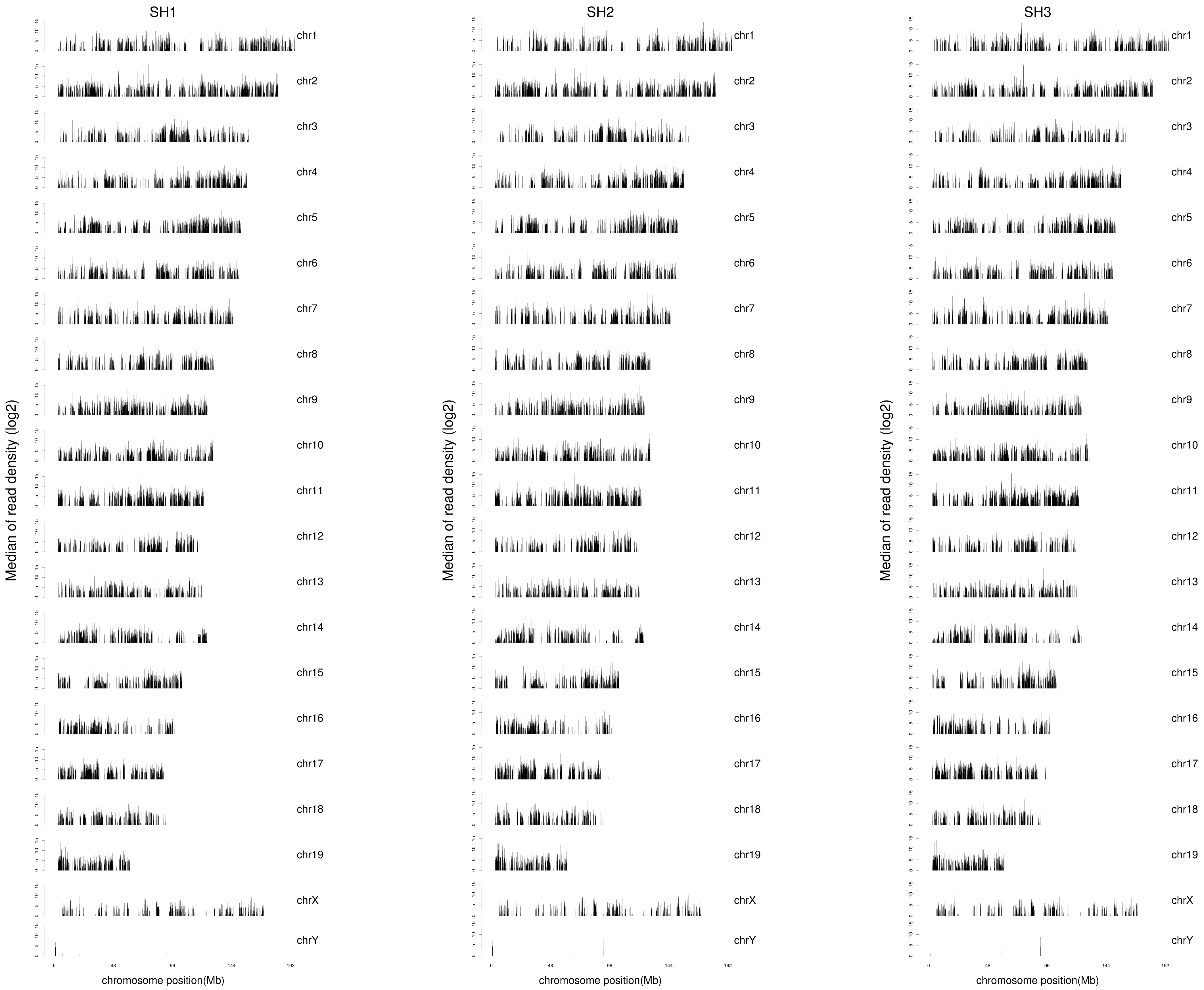

Figure S1: Mapped Read Density vs. Chromosome Position of Sham Muscles

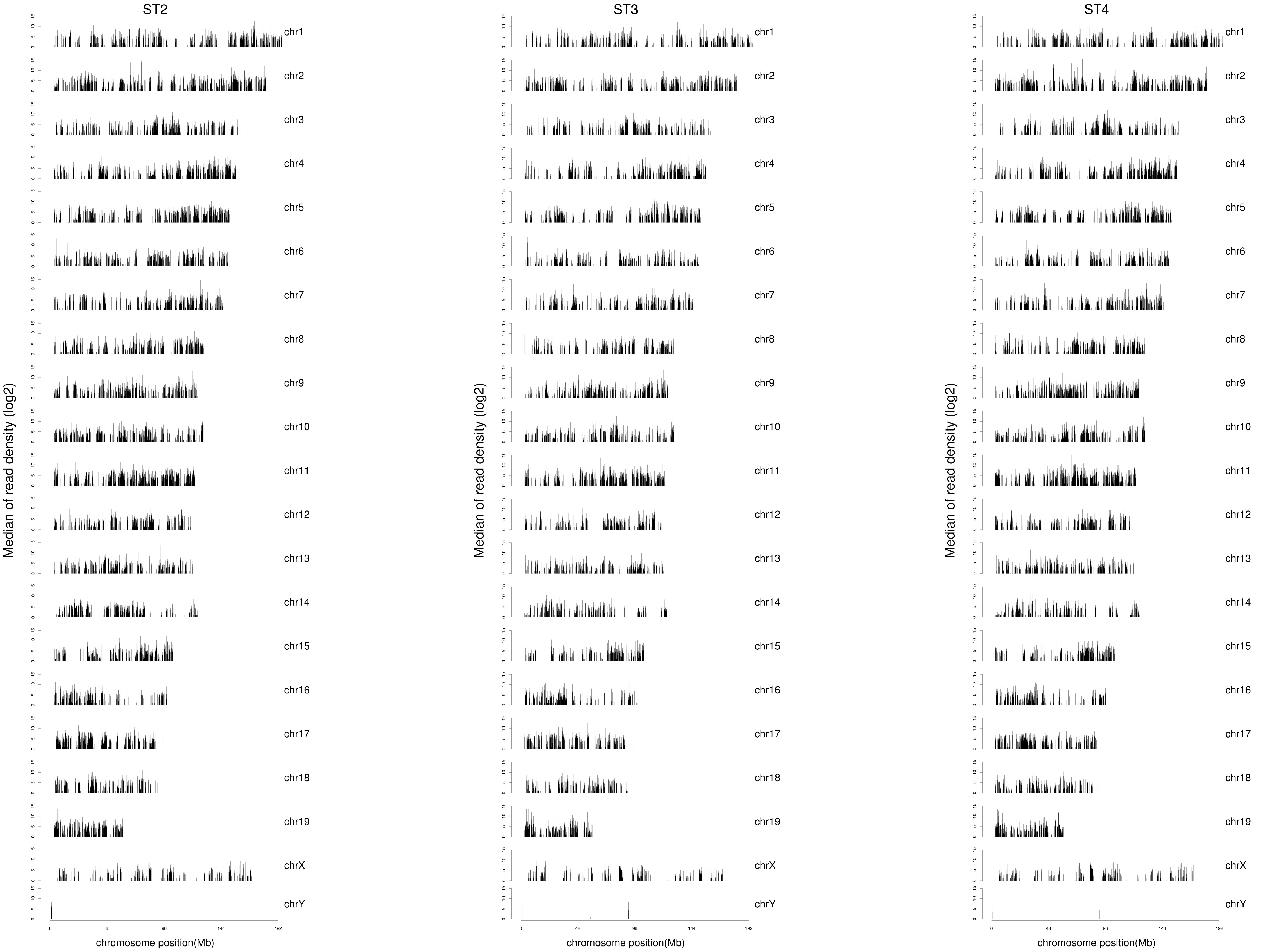

Figure S2: Mapped Read Density vs. Chromosome Position of Stroke Muscles

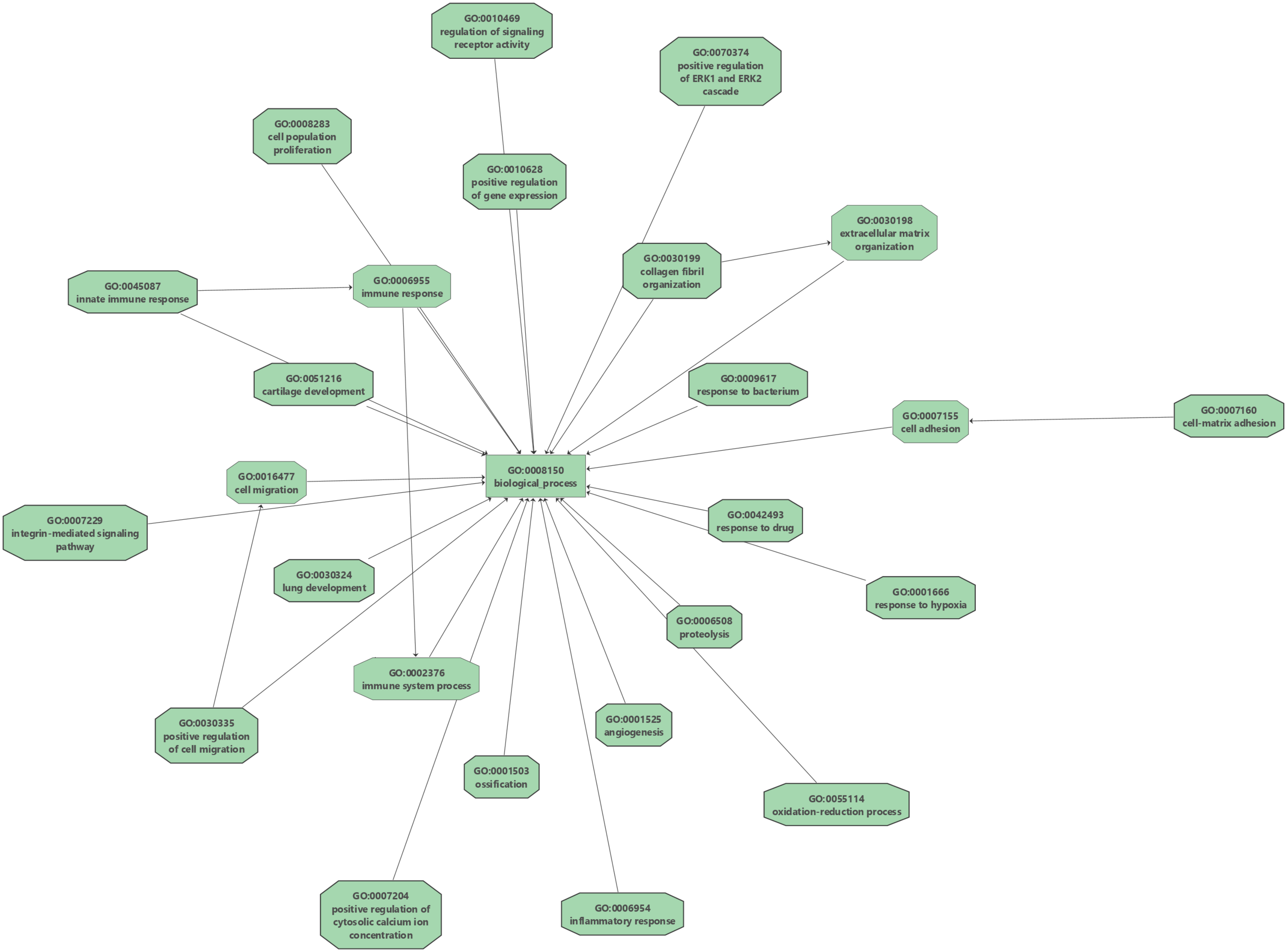

Figure S3: DAG Analysis of GO Terms for “biological process”

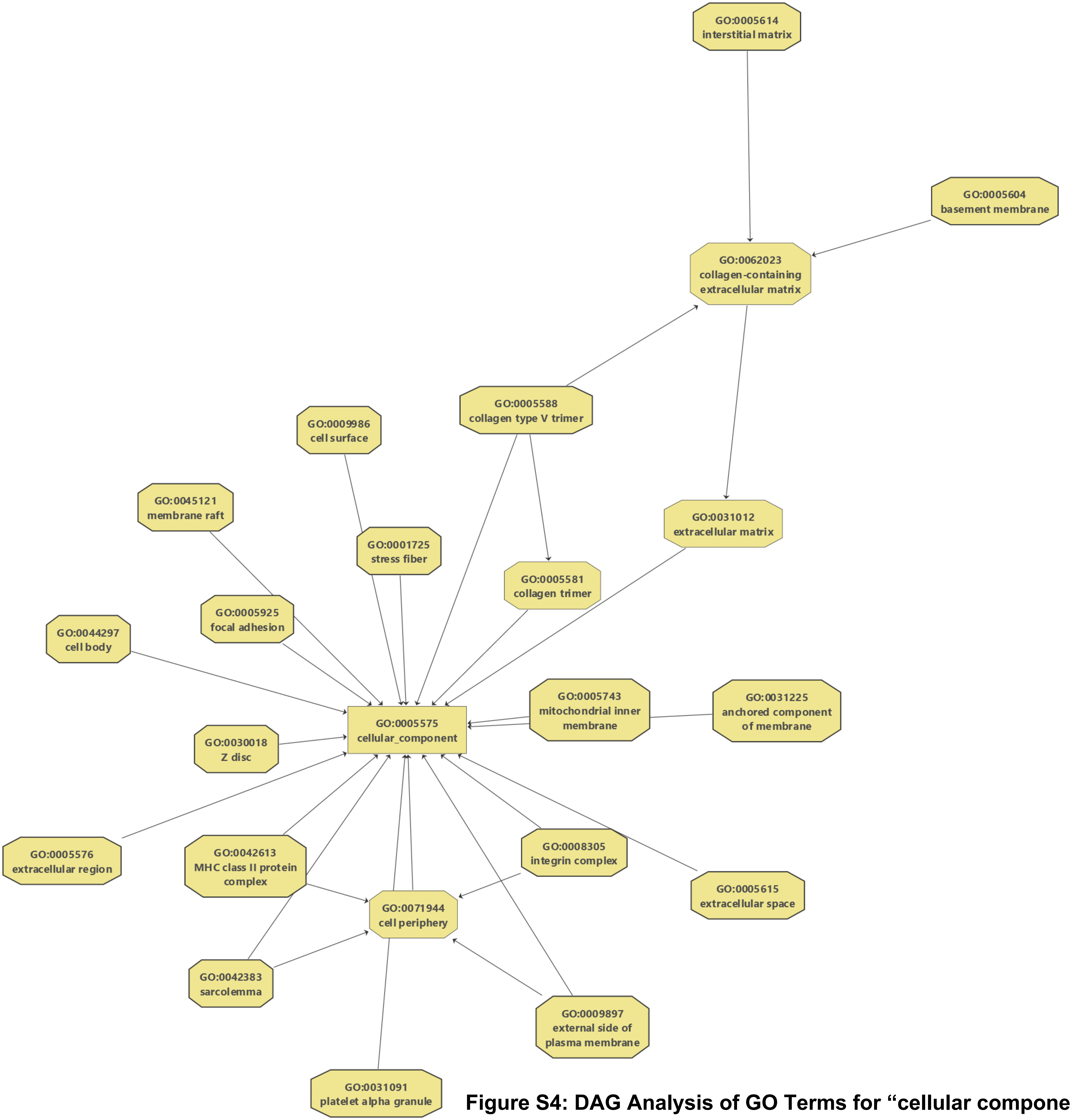

Figure S4: DAG Analysis of GO Terms for “cellular component”

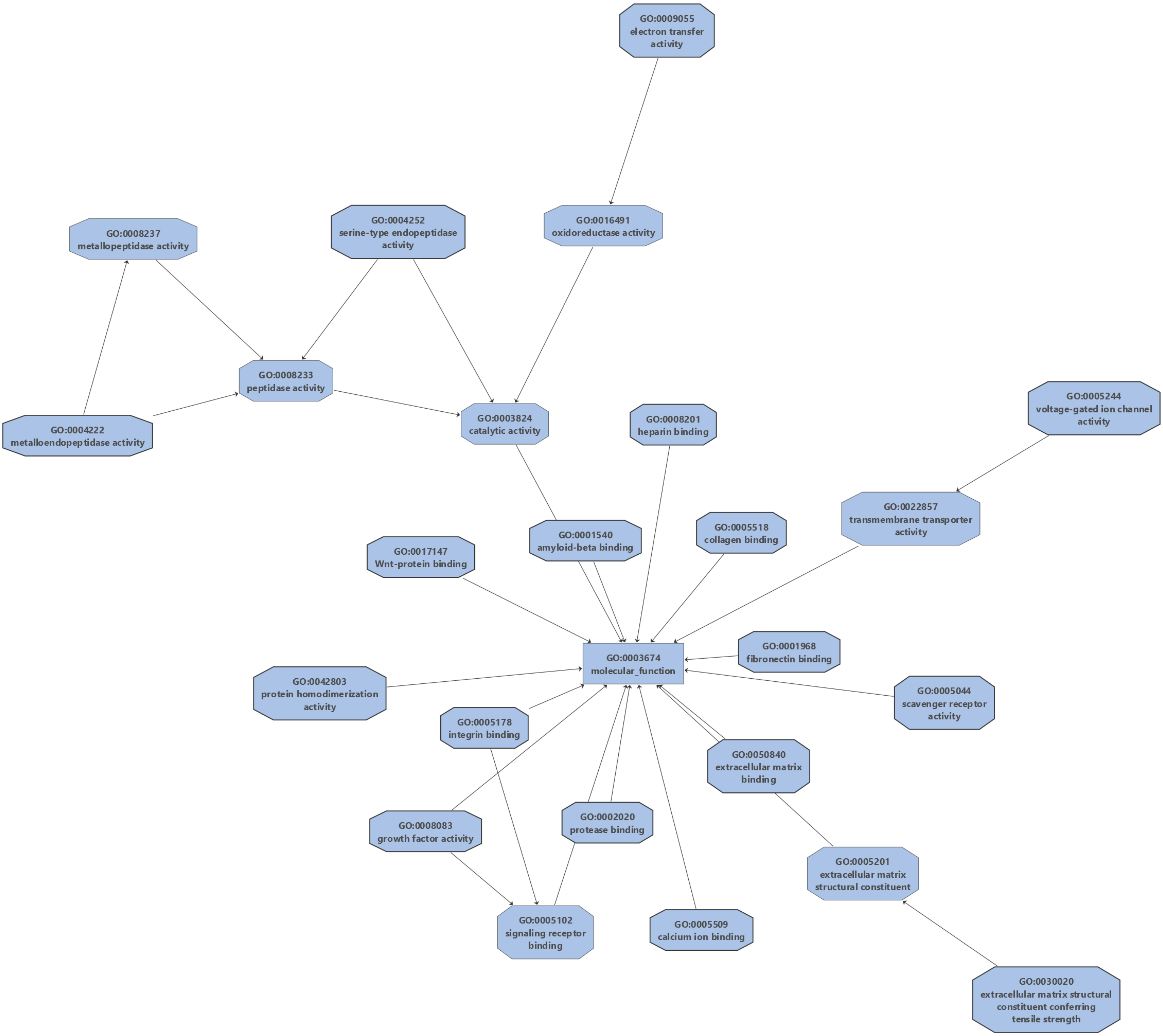

Figure S5: DAG Analysis of GO Terms for “molecular function”
