# Supplementary material for: Transcriptome Analysis of Skeletal Muscle Reveals Altered Proteolytic and Neuromuscular Junction Associated Gene Expressions in a Mouse Model of Cerebral Ischemic Stroke
Source: Genes (Basel). 2020 Jun 30;11(7):726. doi: 10.3390/genes11070726 (PMC7397267; doi:10.3390/genes11070726)
Supplement: Supplementary file 1 [file genes-11-00726-s001.zip › Supplemental Table 1.docx]

Supplemental Table 1

Bioinformatic software and database information.

| **Sample information** | | | | | | | |
| --- | --- | --- | --- | --- | --- | --- | --- |
| Sample ID | SH1 (sham 1) | | SH2 (sham 2) | SH3 (sham 3) | ST2 (stroke 2) | ST3 (stroke 3) | ST4 (stroke 4) |
| **Database** | | | | | | | |
| Genome | <ftp://ftp.ensembl.org/pub/release-96/fasta/mus_musculus/dna/> (v96) | | | | | | |
| lncRNA | <ftp://ftp.sanger.ac.uk/pub/gencode/Gencode_mouse/release_M13/gencode.vM13.long_noncoding_RNAs.gtf.gz> (v13) | | | | | | |
| Gene Orthology (GO) | <http://www.geneontology.org/> (2019.05) | | | | | | |
| KEGG | <http://www.genome.jp/kegg/pathway.html> (2019.05) | | | | | | |
| **Bioinformatics software** | | | | | | | |
| Quality control | | FastQC (vs. 0.10.1) | | | | | |
| Adaptor remove | | Cutadapt (vs. 1.10) | | | | | |
| Mapping | | Hisat (vs. 2.0.4) | | | | | |
| Transcripts assembly | | StringTie (vs. 1.3) | | | | | |
| Differential expression analysis | | R package: edger (N/A) | | | | | |
| GO and KEGG enrichment analysis | | Perl scripts in house (N/A) | | | | | |
| Coding potential | | CPC (Coding Potential Calculator) (vs. 0.9) | | | | | |
| Coding potential | | CNCI (Coding-Non-Coding Index) (vs. 2.0) | | | | | |
