# Supplementary material for: Transcriptome Analysis of Skeletal Muscle Reveals Altered Proteolytic and Neuromuscular Junction Associated Gene Expressions in a Mouse Model of Cerebral Ischemic Stroke
Source: Genes (Basel). 2020 Jun 30;11(7):726. doi: 10.3390/genes11070726 (PMC7397267; doi:10.3390/genes11070726)
Supplement: Supplementary file 1 [file genes-11-00726-s001.zip › Supplemental Table 5 (2fold Upregulated.docx]

Supplemental Table 5

Differentially upregulated genes (≥ 2.0-fold) of post-stroke muscle

| **No** | **Gene** | **Fc** | **Pvalue** | **No** | **Gene** | **Fc** | **Pvalue** |
| --- | --- | --- | --- | --- | --- | --- | --- |
| 1 | Gm3650 | 52.69 | 0.00 | 38 | Kcnh6 | 3.46 | 0.00 |
| 2 | Eif3j2 | 20.21 | 0.00 | 39 | Dlg5;Gm49098 | 3.36 | 0.01 |
| 3 | Gm32939 | 10.47 | 0.05 | 40 | Ctxn3 | 3.36 | 0.00 |
| 4 | Mid1-ps1 | 9.23 | 0.00 | 41 | Pde4b | 3.34 | 0.02 |
| 5 | Sspo | 7.41 | 0.01 | 42 | Arrdc3 | 3.33 | 0.02 |
| 6 | Shroom3 | 6.80 | 0.00 | 43 | Arhgap26;AC110730 | 3.29 | 0.01 |
| 7 | Gm3883 | 6.56 | 0.00 | 44 | Spag5 | 3.22 | 0.00 |
| 8 | Dnajb13 | 6.22 | 0.00 | 45 | Mrgpre | 3.22 | 0.02 |
| 9 | Mfsd4a | 5.94 | 0.00 | 46 | E130218I03Rik | 3.19 | 0.01 |
| 10 | Synpo2 | 5.57 | 0.00 | 47 | Trp53inp1 | 3.17 | 0.03 |
| 11 | Vkorc1l1 | 5.54 | 0.00 | 48 | Mt1 | 3.16 | 0.05 |
| 12 | AC122117 | 5.10 | 0.01 | 49 | Errfi1 | 3.15 | 0.02 |
| 13 | Slc39a14 | 5.04 | 0.03 | 50 | Ttll11 | 3.14 | 0.02 |
| 14 | Itpkc | 4.94 | 0.04 | 51 | Tekt1 | 3.13 | 0.03 |
| 15 | Chrna2 | 4.84 | 0.00 | 52 | Plb1 | 3.11 | 0.04 |
| 16 | Adamts2 | 4.58 | 0.03 | 53 | Sik1 | 3.10 | 0.03 |
| 17 | BB123696 | 4.56 | 0.03 | 54 | Hmgb2 | 3.08 | 0.05 |
| 18 | Gm12240 | 4.53 | 0.02 | 55 | Slc7a5 | 3.05 | 0.00 |
| 19 | Gm10097 | 4.50 | 0.01 | 56 | Kcnv2 | 3.03 | 0.05 |
| 20 | Cdkn1a | 4.47 | 0.02 | 57 | Gm9774 | 3.03 | 0.00 |
| 21 | Eda2r | 4.36 | 0.00 | 58 | Map3k6 | 3.02 | 0.02 |
| 22 | Arl4d | 4.30 | 0.02 | 59 | Egln3 | 3.02 | 0.02 |
| 23 | Tfcp2l1;Gm23497 | 4.25 | 0.01 | 60 | Myog | 3.01 | 0.01 |
| 24 | Acot1;Acot2;Acot4;Acot3 | 4.20 | 0.02 | 61 | Cacna1i | 3.00 | 0.04 |
| 25 | Inhbb | 4.19 | 0.01 | 62 | Eif4ebp1 | 2.97 | 0.01 |
| 26 | Musk | 4.15 | 0.01 | 63 | Sytl1 | 2.96 | 0.05 |
| 27 | Ankrd1 | 4.11 | 0.00 | 64 | Lrrc58;Gm15725 | 2.95 | 0.02 |
| 28 | Rasd2 | 4.10 | 0.00 | 65 | Gm43587 | 2.94 | 0.04 |
| 29 | Angptl4 | 4.06 | 0.00 | 66 | Bach2 | 2.93 | 0.02 |
| 30 | Gnb5;Bcl2l10 | 3.96 | 0.00 | 67 | Gm10382 | 2.89 | 0.00 |
| 31 | Fat2 | 3.90 | 0.02 | 68 | Lcn2 | 2.88 | 0.01 |
| 32 | Mical2 | 3.85 | 0.01 | 69 | Fkbp5 | 2.81 | 0.01 |
| 33 | Gm35330 | 3.83 | 0.01 | 70 | Zkscan6 | 2.80 | 0.03 |
| 34 | Crybb3 | 3.83 | 0.01 | 71 | Kcnk5 | 2.80 | 0.04 |
| 35 | Pfkfb3 | 3.70 | 0.00 | 72 | Pik3r1 | 2.79 | 0.02 |
| 36 | Klf10 | 3.64 | 0.00 | 73 | Hspb7 | 2.76 | 0.01 |
| 37 | Mfhas1 | 3.47 | 0.01 | 74 | Zwint | 2.75 | 0.02 |
| 75 | Gadd45a | 2.71 | 0.04 | 116 | Bex1 | 2.32 | 0.03 |
| 76 | Osgin1 | 2.67 | 0.00 | 117 | Usf3 | 2.32 | 0.01 |
| 77 | Gucd1 | 2.66 | 0.00 | 118 | Tcp11l2 | 2.32 | 0.03 |
| 78 | Ucp3 | 2.65 | 0.01 | 119 | Zfp180 | 2.31 | 0.03 |
| 79 | Gm13357 | 2.62 | 0.03 | 120 | Srrm4 | 2.31 | 0.02 |
| 80 | Pbld1 | 2.59 | 0.03 | 121 | Myo18b | 2.31 | 0.01 |
| 81 | Myc | 2.56 | 0.01 | 122 | Mlycd | 2.30 | 0.01 |
| 82 | Hectd1 | 2.55 | 0.02 | 123 | Gm11661 | 2.30 | 0.04 |
| 83 | Sim2 | 2.54 | 0.00 | 124 | Gm12248 | 2.29 | 0.02 |
| 84 | Sh2d6 | 2.53 | 0.02 | 125 | Eif4a-ps4 | 2.28 | 0.02 |
| 85 | Flnc | 2.52 | 0.01 | 126 | Tnfrsf19 | 2.27 | 0.04 |
| 86 | Rnase13 | 2.52 | 0.03 | 127 | Gan | 2.25 | 0.02 |
| 87 | Tacc2 | 2.51 | 0.03 | 128 | Paip2b | 2.24 | 0.02 |
| 88 | Mospd1 | 2.49 | 0.00 | 129 | 1110012L19Rik | 2.24 | 0.04 |
| 89 | Rsc1a1;Ddi2 | 2.48 | 0.02 | 130 | Tsc22d1 | 2.22 | 0.00 |
| 90 | Map1lc3b | 2.48 | 0.02 | 131 | Igfn1 | 2.22 | 0.03 |
| 91 | Dgkh;Gm48998 | 2.47 | 0.01 | 132 | Rab12 | 2.21 | 0.02 |
| 92 | Slc6a9 | 2.47 | 0.00 | 133 | Atoh7 | 2.21 | 0.04 |
| 93 | Sertad2 | 2.45 | 0.02 | 134 | Coro6 | 2.20 | 0.02 |
| 94 | Rnd1 | 2.45 | 0.04 | 135 | Kctd20 | 2.20 | 0.02 |
| 95 | Ehd3 | 2.45 | 0.01 | 136 | Ube4a | 2.18 | 0.02 |
| 96 | Sgk3 | 2.44 | 0.03 | 137 | 2900026A02Rik | 2.17 | 0.02 |
| 97 | Hacd3 | 2.44 | 0.02 | 138 | Mapk6 | 2.17 | 0.05 |
| 98 | 9130208D14Rik | 2.41 | 0.04 | 139 | Fbxo31 | 2.17 | 0.03 |
| 99 | Mir29b-1 | 2.41 | 0.04 | 140 | Tgm2 | 2.17 | 0.04 |
| 100 | Prune2 | 2.40 | 0.02 | 141 | Ino80b | 2.17 | 0.02 |
| 101 | Kctd1 | 2.39 | 0.01 | 142 | Gclm | 2.16 | 0.03 |
| 102 | Ppp1r27 | 2.39 | 0.03 | 143 | Abcc8 | 2.15 | 0.04 |
| 103 | Kank1 | 2.39 | 0.00 | 144 | Nrap | 2.14 | 0.02 |
| 104 | Cdkn2aipnl | 2.38 | 0.00 | 145 | Gm9826 | 2.14 | 0.04 |
| 105 | Net1 | 2.38 | 0.02 | 146 | AC160336 | 2.14 | 0.04 |
| 106 | Elk4;Slc45a3 | 2.38 | 0.02 | 147 | Eid3 | 2.14 | 0.04 |
| 107 | Chd7 | 2.37 | 0.00 | 148 | Klhl30 | 2.14 | 0.04 |
| 108 | Nap1l1 | 2.37 | 0.00 | 149 | Son | 2.13 | 0.01 |
| 109 | Phospho2 | 2.37 | 0.00 | 150 | Zrsr1 | 2.13 | 0.03 |
| 110 | Clip1 | 2.35 | 0.02 | 151 | Tigd2 | 2.13 | 0.02 |
| 111 | Mir22;Tlcd2 | 2.35 | 0.00 | 152 | Mgea5 | 2.12 | 0.02 |
| 112 | Naca | 2.35 | 0.01 | 153 | Pdlim3 | 2.10 | 0.04 |
| 113 | Slc25a33 | 2.34 | 0.02 | 154 | Gm49602 | 2.09 | 0.04 |
| 114 | Uggt1 | 2.33 | 0.00 | 155 | Sec24a | 2.09 | 0.00 |
| 115 | Sorbs2 | 2.32 | 0.01 | 156 | Naa25 | 2.08 | 0.04 |
| 157 | Ahcy | 2.07 | 0.02 |  |  |  |  |
| 158 | Prob1 | 2.06 | 0.03 |  |  |  |  |
| 159 | Mknk2 | 2.06 | 0.05 |  |  |  |  |
| 160 | Cdkal1 | 2.06 | 0.03 |  |  |  |  |
| 161 | Cblb | 2.06 | 0.02 |  |  |  |  |
| 162 | Lrrc47 | 2.05 | 0.01 |  |  |  |  |
| 163 | Gm38158 | 2.04 | 0.05 |  |  |  |  |
| 164 | Myo18a | 2.03 | 0.03 |  |  |  |  |
| 165 | Zfp414;Pram1 | 2.03 | 0.01 |  |  |  |  |
| 166 | Syngr2;Afmid;Gm20708 | 2.02 | 0.03 |  |  |  |  |
| 167 | Mgll | 2.02 | 0.02 |  |  |  |  |
| 168 | Zcchc24 | 2.01 | 0.01 |  |  |  |  |
| 169 | Nfe2l1 | 2.01 | 0.02 |  |  |  |  |
| 170 | Psmd8 | 2.01 | 0.03 |  |  |  |  |
| 171 | Trp63 | 2.01 | 0.01 |  |  |  |  |
| 172 | Plin5;Plin4 | 2.00 | 0.03 |  |  |  |  |
| 173 | Gbe1 | 2.00 | 0.02 |  |  |  |  |
