# Supplementary material for: Transcriptome Analysis of Skeletal Muscle Reveals Altered Proteolytic and Neuromuscular Junction Associated Gene Expressions in a Mouse Model of Cerebral Ischemic Stroke
Source: Genes (Basel). 2020 Jun 30;11(7):726. doi: 10.3390/genes11070726 (PMC7397267; doi:10.3390/genes11070726)
Supplement: Supplementary file 1 [file genes-11-00726-s001.zip › Supplemental Table 6_correct (2fold downregulated).docx]

Supplemental Table 6

Differentially downregulated genes (≥ 2.0-fold) of post-stroke muscle

| **No** | **Gene** | **FC** | **P value** | **No** | **Gene** | **FC** | **P value** |
| --- | --- | --- | --- | --- | --- | --- | --- |
| 1 | Kctd12 | 0.01 | 0.00 | 38 | Plin1 | 0.10 | 0.00 |
| 2 | Grem2 | 0.02 | 0.00 | 39 | Gm13772 | 0.10 | 0.00 |
| 3 | Timp1 | 0.02 | 0.00 | 40 | AW551984 | 0.11 | 0.00 |
| 4 | Apln | 0.02 | 0.00 | 41 | Wif1 | 0.11 | 0.02 |
| 5 | Sfrp2 | 0.03 | 0.00 | 42 | Rtn4r | 0.11 | 0.00 |
| 6 | Col1a1 | 0.03 | 0.00 | 43 | Mmp3 | 0.11 | 0.04 |
| 7 | Mybph | 0.04 | 0.00 | 44 | Arsi | 0.11 | 0.01 |
| 8 | AC122818 | 0.04 | 0.00 | 45 | Ism1 | 0.11 | 0.00 |
| 9 | Tecta | 0.04 | 0.00 | 46 | Phgdh | 0.12 | 0.00 |
| 10 | Pappa2 | 0.04 | 0.01 | 47 | Col16a1 | 0.12 | 0.00 |
| 11 | Retn | 0.05 | 0.00 | 48 | F830016B08Rik | 0.12 | 0.00 |
| 12 | Gm15429 | 0.05 | 0.00 | 49 | Vcan | 0.12 | 0.00 |
| 13 | Tmem45a | 0.05 | 0.00 | 50 | Kcng4 | 0.12 | 0.01 |
| 14 | Scd1 | 0.05 | 0.00 | 51 | Col4a5 | 0.12 | 0.00 |
| 15 | Col3a1;Gm47302 | 0.05 | 0.00 | 52 | Col14a1 | 0.13 | 0.01 |
| 16 | Aqp4 | 0.05 | 0.00 | 53 | Igfbp5 | 0.13 | 0.00 |
| 17 | Rmi2 | 0.06 | 0.00 | 54 | Elovl6 | 0.13 | 0.00 |
| 18 | Sfrp4 | 0.06 | 0.00 | 55 | Pik3ap1 | 0.13 | 0.01 |
| 19 | Kera | 0.06 | 0.02 | 56 | Actc1 | 0.14 | 0.00 |
| 20 | Gm33543 | 0.06 | 0.00 | 57 | Wfdc1 | 0.14 | 0.00 |
| 21 | Adam12 | 0.06 | 0.00 | 58 | Sema3a | 0.14 | 0.00 |
| 22 | Nrep | 0.06 | 0.00 | 59 | Tnxa | 0.14 | 0.00 |
| 23 | Snora73b | 0.07 | 0.00 | 60 | Cntfr | 0.14 | 0.00 |
| 24 | Mettl21e | 0.07 | 0.00 | 61 | Hp | 0.14 | 0.01 |
| 25 | Tnmd | 0.07 | 0.02 | 62 | Tlr2 | 0.14 | 0.00 |
| 26 | Mfap4 | 0.07 | 0.00 | 63 | Glipr2 | 0.14 | 0.01 |
| 27 | Aldoa | 0.08 | 0.00 | 64 | Tet1 | 0.14 | 0.00 |
| 28 | Ptpn6 | 0.08 | 0.00 | 65 | Ccnf | 0.14 | 0.03 |
| 29 | Col1a2 | 0.08 | 0.00 | 66 | Ccr5 | 0.14 | 0.01 |
| 30 | Postn | 0.09 | 0.00 | 67 | Col11a2 | 0.14 | 0.00 |
| 31 | Folr2 | 0.09 | 0.00 | 68 | Cdo1 | 0.14 | 0.00 |
| 32 | Fam83d | 0.09 | 0.00 | 69 | Igsf10 | 0.15 | 0.00 |
| 33 | Pld5 | 0.09 | 0.04 | 70 | Pi16 | 0.15 | 0.00 |
| 34 | Lrrc52 | 0.09 | 0.00 | 71 | Slc22a3 | 0.15 | 0.01 |
| 35 | Mdga1 | 0.09 | 0.00 | 72 | Pirb | 0.15 | 0.00 |
| 36 | Gm4544 | 0.09 | 0.00 | 73 | Fcrls | 0.15 | 0.02 |
| 37 | Fasn | 0.10 | 0.00 | 74 | Cd84 | 0.15 | 0.01 |
| 75 | Robo2 | 0.15 | 0.00 | 112 | Dact2 | 0.18 | 0.01 |
| 76 | Col11a1 | 0.15 | 0.01 | 113 | Col5a1 | 0.18 | 0.00 |
| 77 | C1qtnf6 | 0.15 | 0.00 | 114 | Cfd | 0.18 | 0.00 |
| 78 | Gm12715 | 0.16 | 0.00 | 115 | Clec11a | 0.19 | 0.00 |
| 79 | Cish | 0.16 | 0.00 | 116 | Fbn1 | 0.19 | 0.00 |
| 80 | Hpse | 0.16 | 0.01 | 117 | Cilp2 | 0.19 | 0.01 |
| 81 | BC049352 | 0.16 | 0.01 | 118 | Igf1 | 0.19 | 0.00 |
| 82 | Cntnap2 | 0.16 | 0.01 | 119 | Foxo6 | 0.19 | 0.00 |
| 83 | H2-DMb1 | 0.16 | 0.01 | 120 | Retnla | 0.19 | 0.01 |
| 84 | Myh7;Myh6 | 0.16 | 0.01 | 121 | Col6a2 | 0.19 | 0.00 |
| 85 | Gm15833 | 0.16 | 0.01 | 122 | Lilrb4a | 0.19 | 0.00 |
| 86 | Adipoq | 0.16 | 0.00 | 123 | Lilr4b | 0.19 | 0.00 |
| 87 | Sv2c | 0.16 | 0.01 | 124 | Tnc | 0.19 | 0.00 |
| 88 | Tfrc | 0.16 | 0.00 | 125 | Fn1 | 0.19 | 0.00 |
| 89 | Gm49311 | 0.16 | 0.01 | 126 | Cmah | 0.19 | 0.01 |
| 90 | Gm48488 | 0.17 | 0.00 | 127 | S100a4;S100a3 | 0.19 | 0.01 |
| 91 | Gm22068 | 0.17 | 0.00 | 128 | Msr1 | 0.19 | 0.01 |
| 92 | Brdt | 0.17 | 0.00 | 129 | Plcd4 | 0.19 | 0.01 |
| 93 | Comp | 0.17 | 0.02 | 130 | Creb3l1 | 0.20 | 0.01 |
| 94 | Adgre1 | 0.17 | 0.00 | 131 | Tnnc1 | 0.20 | 0.05 |
| 95 | Psph | 0.17 | 0.02 | 132 | Dhrs7c | 0.20 | 0.01 |
| 96 | Frzb | 0.17 | 0.00 | 133 | Rcn3 | 0.20 | 0.01 |
| 97 | Chad | 0.17 | 0.01 | 134 | Pcolce;Fbxo24 | 0.20 | 0.00 |
| 98 | Thbs3 | 0.17 | 0.00 | 135 | Gdap1 | 0.20 | 0.02 |
| 99 | Lum | 0.17 | 0.03 | 136 | Col12a1 | 0.20 | 0.00 |
| 100 | Adamts14 | 0.17 | 0.00 | 137 | Col15a1 | 0.20 | 0.00 |
| 101 | Gm14648 | 0.17 | 0.01 | 138 | Rpl10-ps3 | 0.20 | 0.03 |
| 102 | Efhd1 | 0.17 | 0.00 | 139 | Itgb2 | 0.20 | 0.03 |
| 103 | Nckap1l | 0.17 | 0.01 | 140 | Ifit3b | 0.20 | 0.02 |
| 104 | Gpd1 | 0.17 | 0.00 | 141 | Kazald1 | 0.20 | 0.03 |
| 105 | Col9a1 | 0.17 | 0.03 | 142 | Pf4 | 0.20 | 0.01 |
| 106 | AC122252 | 0.17 | 0.00 | 143 | Ctss | 0.20 | 0.00 |
| 107 | Caprin2 | 0.18 | 0.00 | 144 | Aplnr | 0.21 | 0.01 |
| 108 | Glb1l2 | 0.18 | 0.01 | 145 | Fndc4 | 0.21 | 0.02 |
| 109 | Ifi27l2a | 0.18 | 0.01 | 146 | Itm2a | 0.21 | 0.00 |
| 110 | Trim7 | 0.18 | 0.00 | 147 | Cyth4 | 0.21 | 0.01 |
| 111 | Gm49477 | 0.18 | 0.01 | 148 | Slc25a10 | 0.21 | 0.01 |

| 149 | Slc1a4 | 0.21 | 0.04 | 187 | C1rb | 0.23 | 0.01 |
| --- | --- | --- | --- | --- | --- | --- | --- |
| 150 | Gm18066 | 0.21 | 0.02 | 188 | Gm21451 | 0.23 | 0.00 |
| 151 | Col6a1 | 0.21 | 0.00 | 189 | Racgap1 | 0.23 | 0.03 |
| 152 | Ugt1a7c | 0.21 | 0.04 | 190 | Zscan2 | 0.23 | 0.00 |
| 153 | Bmp3 | 0.21 | 0.02 | 191 | Mrgprf | 0.23 | 0.02 |
| 154 | Kcnn2 | 0.22 | 0.00 | 192 | Ptpro | 0.23 | 0.01 |
| 155 | Col6a3 | 0.22 | 0.01 | 193 | Siglec1 | 0.23 | 0.01 |
| 156 | Fmod | 0.22 | 0.02 | 194 | Ndufa12 | 0.24 | 0.00 |
| 157 | Il31ra;Gm3226 | 0.22 | 0.01 | 195 | Akr1b8 | 0.24 | 0.02 |
| 159 | Cadm3;Ackr1 | 0.22 | 0.00 | 196 | Ldha-ps2 | 0.24 | 0.00 |
| 160 | Scd2;Scd3;Mir5114 | 0.22 | 0.00 | 197 | Frk | 0.24 | 0.01 |
| 161 | Pthlh | 0.22 | 0.02 | 198 | Angptl1 | 0.24 | 0.00 |
| 162 | Tmem119 | 0.22 | 0.05 | 199 | Rian | 0.24 | 0.01 |
| 163 | Ptgfr | 0.22 | 0.01 | 200 | Itgam;Gm49368 | 0.24 | 0.00 |
| 164 | Il33 | 0.22 | 0.01 | 201 | Agbl1 | 0.24 | 0.00 |
| 165 | Frem2 | 0.22 | 0.00 | 202 | Serpinf1 | 0.24 | 0.00 |
| 166 | Col5a2 | 0.22 | 0.00 | 203 | Gm9903 | 0.24 | 0.02 |
| 167 | Kctd17 | 0.23 | 0.00 | 204 | Tlr6 | 0.24 | 0.02 |
| 168 | Cd248 | 0.23 | 0.01 | 205 | Lcp1 | 0.24 | 0.01 |
| 169 | Sod3 | 0.23 | 0.00 | 206 | Il1rl2 | 0.24 | 0.00 |
| 170 | Timm22 | 0.23 | 0.00 | 207 | Mrc1 | 0.24 | 0.00 |
| 171 | Pld4 | 0.23 | 0.00 | 208 | Ccdc80 | 0.24 | 0.00 |
| 172 | Ccr2 | 0.23 | 0.04 | 209 | C5ar1 | 0.24 | 0.01 |
| 173 | Alcam | 0.23 | 0.01 | 210 | Ccl6 | 0.24 | 0.01 |
| 174 | Smco1 | 0.23 | 0.01 | 211 | Vat1l | 0.24 | 0.04 |
| 175 | Ndrg4 | 0.23 | 0.00 | 212 | Srebf1;Mir6922 | 0.24 | 0.00 |
| 176 | Dpt | 0.23 | 0.00 | 213 | Prokr1 | 0.24 | 0.02 |
| 177 | Aldh1a2 | 0.23 | 0.02 | 214 | Map2k6 | 0.24 | 0.00 |
| 178 | Ephb2 | 0.23 | 0.02 | 215 | Pcsk5 | 0.24 | 0.01 |
| 179 | Clec3b | 0.23 | 0.02 | 216 | Rbm47 | 0.24 | 0.05 |
| 180 | Olfml1 | 0.23 | 0.02 | 217 | Rasgrp4 | 0.24 | 0.01 |
| 181 | Fstl1 | 0.23 | 0.00 | 218 | Anxa1 | 0.24 | 0.00 |
| 182 | Cd14 | 0.23 | 0.01 | 219 | Lyz1 | 0.25 | 0.00 |
| 183 | Syt12 | 0.23 | 0.01 | 220 | Wnt5a | 0.25 | 0.01 |
| 184 | Lynx1 | 0.23 | 0.00 | 221 | Bdh1 | 0.25 | 0.00 |
| 185 | Dkk2 | 0.23 | 0.01 | 222 | Coro1a | 0.25 | 0.01 |
| 186 | P2ry1 | 0.23 | 0.00 | 223 | Ms4a4a | 0.25 | 0.01 |

| 224 | Tmem25 | 0.25 | 0.04 | 282 | Hs6st2 | 0.28 | 0.03 |
| --- | --- | --- | --- | --- | --- | --- | --- |
| 225 | Dock2 | 0.25 | 0.01 | 283 | Gm14403 | 0.28 | 0.03 |
| 226 | Mettl21c | 0.25 | 0.00 | 284 | Ms4a6c | 0.28 | 0.02 |
| 227 | Tspan11 | 0.25 | 0.00 | 285 | Dpysl3 | 0.28 | 0.01 |
| 228 | Ankmy2 | 0.25 | 0.01 | 286 | Tmem178b | 0.28 | 0.01 |
| 229 | Mif4gd | 0.25 | 0.00 | 287 | Zfp846 | 0.28 | 0.01 |
| 230 | Jph4 | 0.25 | 0.03 | 288 | Casq1 | 0.28 | 0.03 |
| 231 | Itgb6 | 0.25 | 0.04 | 289 | Dpp4 | 0.28 | 0.03 |
| 232 | Abi3bp | 0.25 | 0.01 | 290 | Tirap | 0.28 | 0.01 |
| 233 | Pdpn | 0.25 | 0.00 | 291 | Irf5 | 0.28 | 0.01 |
| 234 | 1700113H08Rik | 0.26 | 0.04 | 292 | Pygl | 0.28 | 0.02 |
| 235 | Tmem218 | 0.26 | 0.02 | 293 | Fcgr2b | 0.28 | 0.01 |
| 236 | Ccr1 | 0.26 | 0.04 | 294 | Ifit3 | 0.28 | 0.02 |
| 237 | Ptger1 | 0.26 | 0.01 | 295 | Nqo2 | 0.28 | 0.02 |
| 238 | AC138790 | 0.26 | 0.02 | 296 | Rnu1a1 | 0.28 | 0.00 |
| 239 | Sema3d | 0.26 | 0.02 | 297 | Slc26a10 | 0.28 | 0.02 |
| 240 | Zfp983;CAAA01141682 | 0.26 | 0.03 | 298 | Magix | 0.28 | 0.00 |
| 241 | Tnni1 | 0.26 | 0.03 | 299 | Ppp1r3b | 0.28 | 0.03 |
| 242 | Gm32036 | 0.26 | 0.01 | 300 | Dhfr | 0.28 | 0.03 |
| 243 | Oaf | 0.26 | 0.01 | 301 | Lgi2 | 0.29 | 0.03 |
| 244 | Slc16a6;Gm25540 | 0.26 | 0.00 | 302 | Igfals | 0.29 | 0.03 |
| 245 | Tyrobp | 0.26 | 0.02 | 303 | Lpar1 | 0.29 | 0.00 |
| 246 | Ackr2 | 0.26 | 0.03 | 304 | Cilp | 0.29 | 0.02 |
| 247 | Myl3 | 0.26 | 0.00 | 305 | Cacnb1 | 0.29 | 0.01 |
| 248 | Pde4a | 0.27 | 0.02 | 306 | Cyb561 | 0.29 | 0.02 |
| 249 | Ugt8a | 0.27 | 0.02 | 307 | Gamt | 0.29 | 0.03 |
| 250 | C3ar1 | 0.27 | 0.01 | 308 | P2ry14 | 0.29 | 0.03 |
| 251 | Emp3 | 0.27 | 0.03 | 309 | Efemp1 | 0.29 | 0.03 |
| 252 | Cpa3 | 0.27 | 0.02 | 310 | Tmem237 | 0.29 | 0.05 |
| 253 | Gm1966 | 0.27 | 0.01 | 311 | Naip6 | 0.29 | 0.03 |
| 254 | Mfap5 | 0.27 | 0.00 | 312 | Thy1 | 0.29 | 0.04 |
| 255 | Crip1 | 0.27 | 0.01 | 313 | F13a1 | 0.29 | 0.01 |
| 256 | Dut | 0.27 | 0.01 | 314 | Scn1b | 0.29 | 0.01 |
| 257 | Olfml2b | 0.27 | 0.01 | 315 | Mgp | 0.29 | 0.00 |
| 258 | Arhgap45 | 0.27 | 0.01 | 316 | Cd80 | 0.29 | 0.05 |
| 280 | Itgb8 | 0.28 | 0.03 | 317 | Prss23 | 0.29 | 0.00 |
| 281 | Kcnf1 | 0.28 | 0.04 | 318 | Emid1 | 0.29 | 0.03 |

| 319 | Fndc1 | 0.29 | 0.01 | 356 | Rita1 | 0.31 | 0.03 |
| --- | --- | --- | --- | --- | --- | --- | --- |
| 320 | H2-Ab1 | 0.29 | 0.02 | 357 | Ak4 | 0.31 | 0.03 |
| 321 | Scrn1 | 0.29 | 0.02 | 358 | Nfam1 | 0.31 | 0.02 |
| 322 | Klhl13 | 0.29 | 0.03 | 359 | Gm9247 | 0.31 | 0.03 |
| 323 | Cpq | 0.29 | 0.02 | 360 | Bgn | 0.31 | 0.00 |
| 324 | Thbs4 | 0.29 | 0.01 | 361 | Plat | 0.31 | 0.01 |
| 325 | Zfp931 | 0.29 | 0.02 | 362 | Gbgt1 | 0.31 | 0.04 |
| 326 | Mir770;Mir1906-1;Gm27300 | 0.30 | 0.00 | 363 | Fads1 | 0.31 | 0.01 |
| 327 | Qpct | 0.30 | 0.03 | 364 | Meox1 | 0.31 | 0.02 |
| 328 | Ighm | 0.30 | 0.01 | 365 | Tph1 | 0.32 | 0.04 |
| 329 | Csf2ra | 0.30 | 0.02 | 366 | Clstn3 | 0.32 | 0.04 |
| 330 | Casp3 | 0.30 | 0.03 | 367 | C1qc | 0.32 | 0.01 |
| 331 | Arhgap30 | 0.30 | 0.01 | 368 | Fam241a | 0.32 | 0.04 |
| 332 | Marcksl1 | 0.30 | 0.04 | 369 | Adcy7 | 0.32 | 0.01 |
| 333 | Chrdl1 | 0.30 | 0.02 | 370 | Atp1b1 | 0.32 | 0.00 |
| 334 | Plek | 0.30 | 0.02 | 371 | Itgbl1 | 0.32 | 0.01 |
| 335 | Cpxm1 | 0.30 | 0.02 | 372 | Anxa2 | 0.32 | 0.00 |
| 336 | Dram1 | 0.30 | 0.02 | 373 | Mmp15 | 0.32 | 0.02 |
| 337 | Col8a1 | 0.30 | 0.02 | 374 | Sorcs2 | 0.32 | 0.03 |
| 338 | Hsd17b12 | 0.30 | 0.02 | 375 | Emilin2 | 0.32 | 0.01 |
| 339 | Egr2 | 0.30 | 0.03 | 376 | Pamr1 | 0.32 | 0.02 |
| 340 | Gsta4 | 0.30 | 0.03 | 377 | Rpl10-ps1 | 0.32 | 0.05 |
| 341 | Fcer1g | 0.30 | 0.01 | 378 | Fsd1l | 0.32 | 0.03 |
| 342 | C1qa | 0.30 | 0.01 | 379 | Fam114a1 | 0.32 | 0.03 |
| 343 | Cercam | 0.30 | 0.04 | 380 | Alpl | 0.32 | 0.02 |
| 344 | Col8a2 | 0.30 | 0.03 | 381 | Best3 | 0.32 | 0.02 |
| 345 | Islr;Islr2 | 0.30 | 0.02 | 382 | Cpxm2 | 0.32 | 0.03 |
| 346 | Ikbke | 0.30 | 0.04 | 383 | Slc37a4 | 0.32 | 0.02 |
| 347 | Ubash3b | 0.31 | 0.04 | 384 | Serpinh1 | 0.32 | 0.01 |
| 348 | Dclk1 | 0.31 | 0.02 | 385 | Tiam1 | 0.32 | 0.03 |
| 349 | Loxl2;Mir6950 | 0.31 | 0.00 | 386 | 0610040J01Rik | 0.32 | 0.05 |
| 350 | Palld | 0.31 | 0.02 | 387 | Zbtb7c | 0.32 | 0.02 |
| 351 | Renbp | 0.31 | 0.04 | 388 | Lgi4 | 0.32 | 0.03 |
| 352 | H2-Eb1 | 0.31 | 0.02 | 389 | Xylt1 | 0.32 | 0.03 |
| 353 | Cotl1 | 0.31 | 0.01 | 390 | C1qb | 0.32 | 0.01 |
| 354 | Gm15953 | 0.31 | 0.04 | 391 | Rnu3b4 | 0.33 | 0.02 |
| 355 | Steap2 | 0.31 | 0.01 | 392 | Tspan6 | 0.33 | 0.03 |

| 393 | Steap3 | 0.33 | 0.01 | 430 | Gpc3 | 0.34 | 0.03 |
| --- | --- | --- | --- | --- | --- | --- | --- |
| 394 | Slc25a1 | 0.33 | 0.05 | 431 | Eml1 | 0.34 | 0.01 |
| 395 | Rab3il1 | 0.33 | 0.03 | 432 | Fcgr3 | 0.34 | 0.04 |
| 396 | Mterf2 | 0.33 | 0.03 | 433 | Itga11 | 0.34 | 0.05 |
| 397 | Ndn | 0.33 | 0.04 | 434 | Cyba | 0.34 | 0.03 |
| 398 | Abhd8 | 0.33 | 0.01 | 435 | Mpz | 0.34 | 0.01 |
| 399 | Fbln1 | 0.33 | 0.03 | 436 | Itgb1bp2 | 0.34 | 0.01 |
| 400 | Apcdd1 | 0.33 | 0.03 | 437 | Gm14005 | 0.34 | 0.05 |
| 401 | Gng11 | 0.33 | 0.02 | 438 | Sept3 | 0.34 | 0.04 |
| 402 | Pmp22 | 0.33 | 0.01 | 439 | Ddah2 | 0.34 | 0.04 |
| 403 | Pcbd2 | 0.33 | 0.03 | 440 | Fitm1 | 0.34 | 0.01 |
| 404 | Tlr13 | 0.33 | 0.05 | 441 | Adamtsl1 | 0.34 | 0.03 |
| 405 | Dhcr7 | 0.33 | 0.04 | 442 | Oasl2 | 0.34 | 0.04 |
| 406 | Rcn1 | 0.33 | 0.02 | 443 | Matn2 | 0.34 | 0.03 |
| 407 | Gprc5c | 0.33 | 0.02 | 444 | Nr4a3 | 0.34 | 0.02 |
| 408 | Lyz2 | 0.33 | 0.00 | 445 | Mtfp1 | 0.34 | 0.02 |
| 409 | Susd2 | 0.33 | 0.02 | 446 | Emp1 | 0.34 | 0.01 |
| 410 | Igfbp6 | 0.33 | 0.01 | 447 | Tnxb | 0.34 | 0.01 |
| 411 | Smim3 | 0.33 | 0.04 | 451 | Mndal;Ifi203 | 0.35 | 0.01 |
| 412 | Cybc1 | 0.33 | 0.02 | 452 | Serpine2 | 0.35 | 0.02 |
| 413 | Ccn3 | 0.33 | 0.03 | 453 | Mcm2 | 0.35 | 0.04 |
| 414 | Pgm1 | 0.33 | 0.03 | 454 | Cnpy4 | 0.35 | 0.04 |
| 415 | Srpx2 | 0.33 | 0.04 | 455 | Pcdh18 | 0.35 | 0.04 |
| 416 | Dock10 | 0.33 | 0.00 | 456 | Lrtm2 | 0.35 | 0.05 |
| 417 | Pde3b | 0.33 | 0.03 | 457 | Adam19 | 0.35 | 0.01 |
| 418 | Gm23444 | 0.34 | 0.04 | 458 | Laptm5 | 0.35 | 0.02 |
| 419 | Bmp1 | 0.34 | 0.00 | 459 | Adamts5 | 0.35 | 0.01 |
| 420 | Fst | 0.34 | 0.02 | 460 | Fermt3 | 0.35 | 0.03 |
| 421 | Syk | 0.34 | 0.01 | 461 | Tspan12 | 0.35 | 0.01 |
| 422 | Slc37a2 | 0.34 | 0.03 | 462 | Grb14 | 0.35 | 0.03 |
| 423 | Mmp19 | 0.34 | 0.02 | 463 | Ldhb | 0.35 | 0.01 |
| 424 | Id2 | 0.34 | 0.04 | 464 | Alox5ap | 0.35 | 0.04 |
| 425 | En1 | 0.34 | 0.05 | 465 | Gm22154 | 0.35 | 0.04 |
| 426 | Adcy1 | 0.34 | 0.03 | 466 | Cybb | 0.35 | 0.05 |
| 427 | Mfap3l | 0.34 | 0.04 | 467 | Lgals3 | 0.35 | 0.01 |
| 428 | Ccnd1 | 0.34 | 0.01 | 468 | Col18a1 | 0.35 | 0.02 |
| 429 | Mafa | 0.34 | 0.04 | 469 | S100a11 | 0.35 | 0.03 |

| 470 | Mmp2 | 0.35 | 0.00 | 507 | Angptl2 | 0.37 | 0.04 |
| --- | --- | --- | --- | --- | --- | --- | --- |
| 471 | Gm14410 | 0.36 | 0.05 | 508 | Myof | 0.37 | 0.01 |
| 472 | Fxyd6 | 0.36 | 0.01 | 509 | Themis2 | 0.37 | 0.05 |
| 473 | Slc25a35 | 0.36 | 0.04 | 510 | Fads2 | 0.37 | 0.03 |
| 474 | A130010J15Rik;Irf6 | 0.36 | 0.03 | 511 | Bace2 | 0.37 | 0.04 |
| 475 | Adra1a | 0.36 | 0.04 | 512 | Gpm6b | 0.37 | 0.02 |
| 476 | Lama4 | 0.36 | 0.00 | 513 | Pola1 | 0.37 | 0.05 |
| 477 | Hspa1b | 0.36 | 0.04 | 514 | Umps | 0.37 | 0.03 |
| 478 | Sparc | 0.36 | 0.01 | 515 | S1pr2 | 0.37 | 0.04 |
| 479 | Mrc2 | 0.36 | 0.02 | 516 | Zfp385b | 0.37 | 0.02 |
| 480 | C3 | 0.36 | 0.04 | 517 | Pcolce2 | 0.37 | 0.04 |
| 481 | Ndufa4l2 | 0.36 | 0.02 | 518 | Aspn | 0.38 | 0.02 |
| 482 | Tgfbi | 0.36 | 0.01 | 519 | Coq7 | 0.38 | 0.02 |
| 483 | Lgmn | 0.36 | 0.01 | 520 | Eno3 | 0.38 | 0.02 |
| 484 | Enpep | 0.36 | 0.02 | 521 | Cav3 | 0.38 | 0.03 |
| 485 | C4b | 0.36 | 0.02 | 522 | Cxcl14 | 0.38 | 0.02 |
| 486 | Homer2 | 0.36 | 0.01 | 523 | Csf1r | 0.38 | 0.03 |
| 487 | Ttll12 | 0.36 | 0.03 | 524 | Adgrd1 | 0.38 | 0.01 |
| 488 | Chpf | 0.36 | 0.02 | 525 | Il17ra | 0.38 | 0.04 |
| 489 | Alox5 | 0.36 | 0.04 | 526 | Ccn2 | 0.38 | 0.00 |
| 490 | Cacna1g | 0.36 | 0.03 | 527 | Sdc2 | 0.38 | 0.02 |
| 491 | Lamb1 | 0.37 | 0.00 | 528 | Enpp1 | 0.38 | 0.05 |
| 492 | Dse | 0.37 | 0.03 | 529 | S1pr3 | 0.38 | 0.03 |
| 493 | Sgsh | 0.37 | 0.03 | 530 | Cdc42ep2 | 0.38 | 0.04 |
| 494 | Tomm40l | 0.37 | 0.01 | 531 | Nid2 | 0.38 | 0.02 |
| 495 | Tppp3 | 0.37 | 0.01 | 532 | Ppic | 0.38 | 0.04 |
| 496 | Apobec2 | 0.37 | 0.03 | 533 | Ace | 0.38 | 0.01 |
| 497 | Cptp | 0.37 | 0.05 | 534 | Ecm2 | 0.38 | 0.03 |
| 498 | Ramp1 | 0.37 | 0.01 | 535 | Fat1 | 0.38 | 0.01 |
| 499 | Rxrg | 0.37 | 0.05 | 536 | Klhl33 | 0.38 | 0.01 |
| 500 | Pltp | 0.37 | 0.03 | 537 | Rap1gap2 | 0.39 | 0.03 |
| 501 | Vopp1 | 0.37 | 0.04 | 538 | Myoz3 | 0.39 | 0.03 |
| 502 | C2;Cfb;Gm20547 | 0.37 | 0.03 | 539 | B4galt6 | 0.39 | 0.05 |
| 503 | Fzd2 | 0.37 | 0.04 | 540 | Ckmt2 | 0.39 | 0.01 |
| 504 | Tmem9 | 0.37 | 0.05 | 541 | Fgl2 | 0.39 | 0.04 |
| 505 | Sel1l3 | 0.37 | 0.04 | 542 | Casq2 | 0.39 | 0.01 |
| 506 | Gm2606 | 0.37 | 0.03 | 543 | Akr1b10 | 0.39 | 0.01 |

| 544 | Gm24407 | 0.39 | 0.02 | 581 | Cyp2e1 | 0.40 | 0.04 |
| --- | --- | --- | --- | --- | --- | --- | --- |
| 545 | Rab3a | 0.39 | 0.03 | 582 | Ecm1;Mir7014 | 0.40 | 0.01 |
| 546 | Atp10a | 0.39 | 0.05 | 583 | Cyc1 | 0.40 | 0.01 |
| 547 | Cd34 | 0.39 | 0.01 | 584 | Hspa12a | 0.40 | 0.03 |
| 548 | Gm24265 | 0.39 | 0.02 | 585 | Ldha | 0.40 | 0.04 |
| 549 | Clcn5 | 0.39 | 0.04 | 586 | Ak1 | 0.41 | 0.03 |
| 550 | Fam102a | 0.39 | 0.03 | 587 | Antxr2 | 0.41 | 0.05 |
| 551 | Antxr1 | 0.39 | 0.04 | 588 | Rbms3 | 0.41 | 0.03 |
| 552 | Col5a3 | 0.39 | 0.01 | 589 | Faap100 | 0.41 | 0.03 |
| 553 | Mpnd | 0.39 | 0.02 | 590 | Sulf2 | 0.41 | 0.03 |
| 554 | Gbp10;Gm43302;Gbp6 | 0.39 | 0.03 | 591 | Cd200 | 0.41 | 0.02 |
| 555 | Fam126a | 0.40 | 0.02 | 592 | Myoc | 0.41 | 0.03 |
| 556 | Cd74;Mir5107 | 0.40 | 0.05 | 593 | Snord17 | 0.41 | 0.03 |
| 557 | Tent5a | 0.40 | 0.01 | 594 | Cd44 | 0.41 | 0.02 |
| 558 | Paqr9 | 0.40 | 0.02 | 595 | Tstd3 | 0.41 | 0.04 |
| 559 | Ifi205;Ifi204 | 0.40 | 0.04 | 596 | H60b;Raet1e;Raet1d | 0.41 | 0.04 |
| 560 | Lhfp | 0.40 | 0.02 | 597 | Pld2 | 0.41 | 0.03 |
| 561 | Serping1 | 0.40 | 0.02 | 598 | Acly | 0.41 | 0.03 |
| 562 | Sesn3 | 0.40 | 0.01 | 599 | Metrn;Fam173a;AC134908 | 0.42 | 0.04 |
| 563 | Unc93b1 | 0.40 | 0.04 | 600 | Lima1 | 0.42 | 0.02 |
| 564 | Tpi1 | 0.40 | 0.03 | 601 | S100a10 | 0.42 | 0.04 |
| 565 | Sirt3 | 0.40 | 0.02 | 602 | Myl9 | 0.42 | 0.02 |
| 566 | Rp2 | 0.40 | 0.05 | 603 | Bcs1l | 0.42 | 0.05 |
| 567 | Ms4a4d | 0.40 | 0.03 | 604 | Gatad1 | 0.42 | 0.02 |
| 568 | Tagln2 | 0.40 | 0.01 | 605 | Mapk8ip1 | 0.42 | 0.05 |
| 569 | S100a6 | 0.40 | 0.01 | 606 | Gm23804 | 0.42 | 0.04 |
| 570 | Gpr153 | 0.40 | 0.03 | 607 | C1s1 | 0.42 | 0.02 |
| 571 | Axl | 0.40 | 0.02 | 608 | Sparcl1 | 0.42 | 0.02 |
| 572 | Cnn2 | 0.40 | 0.04 | 609 | Gm22634 | 0.42 | 0.05 |
| 573 | Apba1 | 0.40 | 0.05 | 610 | Ltbp4 | 0.42 | 0.02 |
| 574 | Ggcx | 0.40 | 0.03 | 611 | Sulf1 | 0.42 | 0.01 |
| 575 | Smoc2 | 0.40 | 0.02 | 612 | Rcbtb2 | 0.42 | 0.04 |
| 576 | Fndc5 | 0.40 | 0.01 | 613 | Tagln | 0.43 | 0.03 |
| 577 | Col4a3 | 0.40 | 0.04 | 614 | Mxra8 | 0.43 | 0.02 |
| 578 | Slc1a5 | 0.40 | 0.02 | 615 | Pon3 | 0.43 | 0.03 |
| 579 | Gm49450;Tuba1a;Tuba1b | 0.40 | 0.02 | 616 | Mettl9 | 0.43 | 0.03 |
| 580 | Myoz2 | 0.40 | 0.05 | 617 | Timmdc1 | 0.43 | 0.03 |

| 618 | Pcsk6;Snrpa1 | 0.43 | 0.03 | 656 | Cycs | 0.45 | 0.03 |
| --- | --- | --- | --- | --- | --- | --- | --- |
| 619 | Stab1 | 0.43 | 0.02 | 657 | Thbs2 | 0.45 | 0.04 |
| 620 | Hmcn2 | 0.43 | 0.03 | 658 | Fbln2 | 0.45 | 0.05 |
| 621 | Trpc3 | 0.43 | 0.02 | 659 | Dnmt1 | 0.46 | 0.05 |
| 622 | Atp5g3 | 0.43 | 0.02 | 660 | Zfp827 | 0.46 | 0.04 |
| 623 | Vim | 0.43 | 0.03 | 661 | Evc | 0.46 | 0.04 |
| 624 | Ifngr2 | 0.43 | 0.04 | 662 | Fam57b | 0.46 | 0.05 |
| 625 | Vtn | 0.43 | 0.02 | 663 | Dnajc15 | 0.46 | 0.03 |
| 626 | Nbl1 | 0.43 | 0.02 | 664 | Ndufb6 | 0.46 | 0.02 |
| 627 | Mrps36 | 0.43 | 0.04 | 665 | Kcnj8 | 0.46 | 0.03 |
| 628 | Itih5 | 0.43 | 0.04 | 666 | Acta2 | 0.46 | 0.04 |
| 629 | Mdh1 | 0.43 | 0.02 | 667 | Slc25a4 | 0.46 | 0.03 |
| 630 | Anxa5 | 0.43 | 0.02 | 668 | Abhd11 | 0.46 | 0.03 |
| 631 | Ccnd2 | 0.43 | 0.05 | 669 | Atp5g1 | 0.46 | 0.03 |
| 632 | Fam198b | 0.43 | 0.04 | 670 | Sh3bgr | 0.47 | 0.04 |
| 633 | Loxl1 | 0.43 | 0.03 | 671 | Pam | 0.47 | 0.04 |
| 634 | Clu | 0.43 | 0.04 | 672 | Adamtsl5 | 0.47 | 0.05 |
| 635 | Cox7a1 | 0.43 | 0.02 | 673 | Kcnb1 | 0.47 | 0.04 |
| 636 | Fopnl | 0.44 | 0.05 | 674 | Prg4 | 0.47 | 0.04 |
| 637 | Cox7a2 | 0.44 | 0.03 | 675 | Srebf2 | 0.47 | 0.03 |
| 638 | Rgs4 | 0.44 | 0.04 | 676 | Podxl | 0.47 | 0.03 |
| 639 | Nrp1;Mir1903 | 0.44 | 0.02 | 677 | Kank2 | 0.47 | 0.04 |
| 640 | Pitpnc1 | 0.44 | 0.02 | 678 | Scn7a | 0.47 | 0.05 |
| 641 | Rnase4;Ang | 0.44 | 0.02 | 679 | Parva | 0.48 | 0.03 |
| 642 | Ldlr | 0.44 | 0.01 | 680 | Ndufa8 | 0.48 | 0.04 |
| 643 | Mb | 0.44 | 0.03 | 681 | Plau | 0.48 | 0.05 |
| 644 | Slc25a19 | 0.44 | 0.04 | 682 | Dph3 | 0.48 | 0.05 |
| 645 | Lbp | 0.44 | 0.05 | 683 | Lrp1 | 0.48 | 0.04 |
| 646 | Nid1 | 0.44 | 0.02 | 684 | Dpysl2 | 0.48 | 0.05 |
| 647 | Csf1 | 0.44 | 0.03 | 685 | Gm26712;Pyurf | 0.48 | 0.05 |
| 648 | Egflam | 0.44 | 0.03 | 686 | Adk | 0.48 | 0.05 |
| 649 | Rftn1 | 0.44 | 0.03 | 687 | Man1a | 0.48 | 0.05 |
| 650 | Plxdc1 | 0.44 | 0.05 | 688 | Minos1 | 0.49 | 0.05 |
| 651 | Sdhd | 0.45 | 0.03 | 689 | Ndufb10 | 0.49 | 0.05 |
| 652 | Mrpl2 | 0.45 | 0.03 | 690 | Gm10039 | 0.49 | 0.03 |
| 653 | Mlf1 | 0.45 | 0.03 | 691 | Mrpl42 | 0.49 | 0.04 |
| 654 | Pdgfra;Mir7025 | 0.45 | 0.03 | 692 | Steap4 | 0.49 | 0.04 |
| 655 | Idh3a | 0.45 | 0.04 |  |  |  |  |
